# Bridging the gap: coordinating equity and efficiency in older people care resource allocation in China

**DOI:** 10.1186/s12877-024-04696-w

**Published:** 2024-02-16

**Authors:** Liangwen Zhang, Linjiang Wei, Wenzheng Zhang, Ya Fang

**Affiliations:** 1 State Key Laboratory of Molecular Vaccinology and Molecular Diagnostics, School of Public Health, Xiamen University, Xiamen, China; 2 Key Laboratory of Health Technology Assessment of Fujian Province, School of Public Health, Xiamen University, Xiamen, China

**Keywords:** Older people care resource, Resource allocation, Equity, Efficiency, China

## Abstract

**Background:**

With the increasing global aging population, how to allocate older people care resources reasonably has become an increasingly urgent international issue. China, as the largest developing country, has made many efforts to actively respond to the challenges of an aging population. However, there are still problems with uneven allocation of older people care resources and low efficiency of allocation. Therefore, this study evaluates the regional differences and dynamic evolution of the equity and efficiency of older people care resource allocation in China from 2009 to 2020, and explores ways to change the current situation.

**Methods:**

The data used in this study were derived from the “China Statistical Yearbook” and the “China Civil Affairs Statistical Yearbook” for the period of 2010–2021. Firstly, the equity of older people care resource allocation was measured using the Gini coefficient, the Theil index, the Older People Care Resource Density Index, and the Older People Care Resource Agglomeration Degree. Secondly, the dynamic Slack-Based Measure data envelopment analysis method was adopted to evaluate efficiency. Lastly, the Z-score is used to normalize the equity index and perform classification matching with the efficiency value. Spatial autocorrelation analysis and hotspot analysis were conducted using GIS technology to examine the dynamic evolution process of older people care resource allocation equity and efficiency, as well as their spatial distribution patterns and coordination across provinces from 2009 to 2020.

**Results:**

The equity analysis showed that the spatial distribution of various types of older people care resources was uneven, and the differences were mainly due to internal differences within each region, with the largest equity differences observed in western provinces. Currently, older people care resources are mainly concentrated in eastern regions, while the total amount of older people care resources in western regions and some central regions is relatively small, which cannot meet the older people care needs of residents. The efficiency analysis results showed that the efficiency of older people care resource allocation has been improving over the past 12 years, and in 2020, 77.42% of provinces were located on the efficiency frontier with an average efficiency value of 0.9396. Finally, the coordination analysis results showed that there were significant spatiotemporal differences in the equity and efficiency of older people care resources allocation.

**Conclusion:**

With the development of society and economy, the total amount and service capacity of older people care resources in China have greatly improved. However, there are still significant spatiotemporal differences in the equity and efficiency of older people care resource allocation. The development of older people care services in central and eastern provinces is unbalanced, and there is a polarization trend in terms of equity and efficiency of older people care resource allocation. Most provinces in western regions face the dual dilemma of inadequate older people care resources and low utilization efficiency. It is recommended that policymakers comprehensively consider population and geographic factors in different provinces, establish relevant allocation standards according to local conditions, improve the redistribution system, and focus on increasing the total amount of older people care resources in underdeveloped provinces while promoting resource flow.

**Supplementary Information:**

The online version contains supplementary material available at 10.1186/s12877-024-04696-w.

## Background

As more countries around the world begin facing the complex health challenges brought about by aging populations [1], with the number of those aged 60 years or over predicted to rise from 962 million in 2017 to 2.1 billion in 2050 and 3.1 billion in 2100 [2]. Older people care resources (OPCR) have garnered widespread attention and become an urgent issue for international research [3]. As a developing country with the largest elderly population, China’s aging process has been deepening continuously, which is characterized by the increasing number of older people, the rising demand for healthcare services, and the inadequate supply of OPCRs. Especially after the outbreak of COVID-19, older people have become a key care target due to their weakened immune system, leading to a continuous increase in China’s demand for older people care services [4]. The rapid development of China’s older people care industry can be traced back to 1999 when the government launched over 100 policies related to older people care services from 1999 to 2012 [5]. This marked a significant developmental leap from “older people care services” to “older people care service system” to “social older people care service system”. Market-oriented older people care services such as home-based care, institutional care, and community-based care emerged and gained popularity [6]. Since 2013, the Chinese government has launched over 150 policies related to older people care services, and the multi-level older people care service system has entered a golden period of development [5]. (Note: OPCR stands for “older people care resource” and will be used in subsequent mentions).

After more than 70 years of development, China’s older people care service system has made great progress. However, due to China’s vast geographical expanse, enormous population, and significant socioeconomic disparities between regions, uneven levels of government investment, and limitations on inter-regional resource mobility, OPCR such as personnel, finances, and goods are spatially mismatched, unevenly distributed, and lacking in equity [7], with varying levels of service efficiency and quality [8]. The 2022 State Council’s plan for the development of the national aging industry and older people care service system during the 14th Five-Year Plan period emphasizes the need to expand the supply capacity of older people care services, improve the rational allocation and balanced layout of OPCR, increase the coverage and convenience of older people care services, and promote the high-quality development of older people care services. Meanwhile, the important concept of balanced regional development of older people care services was proposed in the government work report for the National People’s Congress and Chinese People’s Political Consultative Conference in 2023, promoting the construction of a Chinese-style “more equitable and sustainable” older people care service system [9, 10].

However, most studies have focused on describing the allocation of specific types of resources in certain provinces, autonomous regions, or municipalities [7, 11]. For example, Yin Kongyang et al. [12] pioneered research on the efficiency of OPCR allocation in developed cities. There is a scarcity of research that analyzes the spatio-temporal variations in both equity and efficiency of older people care facility resource allocation at a national level. Furthermore, existing research approaches mostly tend to independently analyze the equity and efficiency of OPCR allocation [13–16], with few studies integrating both aspects into a comprehensive evaluation framework. For instance, Song Qi [17] evaluated the older people service resource allocation in counties of Yunnan Province separately based on efficiency and equity, yielding regional-specific conclusions but lacking generalizability and extrapolation. Peixi Wang et al. [10] combined the efficiency and equity of resource allocation and conducted a coordination assessment of OPCRs in Guangdong Province using a two-dimensional quadrant diagram. However, due to the use of cross-sectional data, they were unable to explore the temporal changes in the efficiency and equity of OPCR allocation, thereby limiting the government’s access to dynamic evolutionary information.

Based on a review and summary of relevant literature, the paper begins by comprehensively reviewing the current state of research on equity and efficiency in OPCR allocation. It then established the importance of evaluating these dimensions longitudinally and spatially in the Chinese context. To assess equity, a range of established indicators were selected including the Gini coefficient, Theil index, resource density index and agglomeration degree. These captured distribution patterns from the perspectives of both population and geographical factors. Lorenz curves were also utilized to illustrate equity trends visually from 2009–2020. Efficiency was examined through a novel dynamic slack-based measure data envelopment analysis model. This accounted for input-output flexibility and intertemporal relationships over the 12-year study period. Relevant labor, capital and output metrics were identified based on economic theory. Equity and efficiency results were then presented separately, identifying different characteristics. A matching analysis was conducted to explore coordination between the two dimensions based on standardized scores. Spatial tools like LISA clustering further revealed heterogeneous patterns across regions. This generated novel insights into how statuses converged or diverged in different provincial contexts. The analysis yielded important conclusions on unbalanced development and polarization trends nationally. It was found that while resources and services expanded greatly, significant spatial mismatches remain—particularly in western China.

This study makes several important innovations. First, we develop an original analytical framework integrating the traditionally separate dimensions of equity and efficiency. By evaluating their coordination longitudinally and spatially, our approach yields novel insight into resource allocation dynamics. Second, we apply a comprehensive set of quantitative metrics to systematically characterize trends across China’s diverse regions between 2009–2020. This 12-year examination generates a more holistic and up-to-date picture than prior cross-sectional analyses. Third, our use of spatial statistics uncovered meaningful clustering patterns that advances understanding of intra- and inter-regional disparities. Policymakers can leverage these findings to more appropriately target underserved localities.

## Methods

### Data resources, regional division, and statistical analysis

The data used in this study are all from official publications. The data used for the equity analysis come from the “China Statistical Yearbook”, while the input-output indicators used in the Data Envelopment Analysis (DEA) analysis come from the “China Civil Affairs Statistical Yearbook” and the statistical yearbooks of various provinces. Due to data availability, this study selected the period from 2009 to 2020 and used a series of longitudinal indicators to evaluate the efficiency and equity of China’s OPCR allocation. As this study aimed to evaluate trends at the national level across this time period, we sampled all available provincial-level data for mainland China during these years. Specifically, this study included data for all 31 provincial-level divisions which included 23 provinces, 5 autonomous regions, and 4 direct-controlled municipalities. By encompassing the entire geographical range and population of China, this sampling approach allowed for a truly nationwide analysis. This study assessed equity and efficiency trends without bias by considering each province as an equal unit, regardless of size or development level. Additionally, China was divided into four regions, namely, western, central, eastern, and northeastern regions, according to the level of economic development and geographical location [18].

The analysis tools used in this study include the STATA MP17 software for calculating equity indicators such as the Gini coefficient and Theil index and drawing Lorenz curves, data envelopment analysis (DEA) using the Python language in Anaconda, and ArcGIS 10.8 for drawing maps of the distribution of OPCRs.

### Indicators of equity and efficiency

As there is limited research on the equity of OPCRs, this study drew on the analysis of healthcare resources, which share high similarity with OPCRs, and selected four indicators for equity analysis: the number of older people care institutions, the number of active employees, the number of beds in older people care institutions, and the government financial allocation received by the older people care industry [10, 19].

In the DEA analysis, this paper combines previous research and the production factor theory in economics to select relevant variables from the labor and capital perspectives. Among the input indicators, labor input includes the number of employees, social workers, managers, and skilled professionals; capital input includes the total number of older people care institutions, fixed assets, year-end beds, building area, and operating profits from the previous year [20, 19, 21–26]. Older people care institutions include all social service institutions that provide accommodation for the older people in both urban and rural areas. The output indicators include the annual operating profit, the number of residents in the institution during the year (including self-care, assisted care, and nursing care), the total days of residence during the year, and the number of rehabilitation and medical outpatient visits [24, 26–30]. The annual operating profit is a transfer variable in the model and serves as both the output in the first year and input in the second year. Generally, the number of Decision Making Units (DMU) is required to be twice the sum of the input variables and output variables to obtain the discriminative power of DEA [31], which is met in this study (31 > 2 × (5 + 6)).

In addition, for missing values in each indicator, this study used machine learning techniques, specifically the random forest imputation method, to fill in the missing values.

### Statistical analysis

#### Equity analysis

##### Gini coefficient (G) and Lorenz curve

These are two widely used economic methods for evaluating the equity of resource allocation [19, 21, 26]. This study introduces them to reflect the degree of equity of OPCR allocation among provinces. The Lorenz curve divides OPCRs (the number of older people care institutions, the number of beds, the number of older people care service personnel, and the financial allocation received by the older people care industry) into several levels based on different populations or regions, arranged from smallest to largest. The cumulative percentages of population (geographical area) and OPCRs are calculated, and the corresponding percentages are plotted on the x and y axes, respectively. The Lorenz curve is obtained by smoothly connecting the points. The closer the curve is to the absolute equity line (diagonal line), the fairer the allocation of OPCRs is; the farther the curve is from the absolute equity line, the more unfair the allocation of OPCRs is.

The Gini coefficient G is a statistical indicator calculated based on the Lorenz curve, which is used to measure the degree of equity of social income or resource allocation. It is equal to the ratio of the area between the absolute equity line and the Lorenz curve to the area of the right-angled triangle below the absolute equity line.

$$G=\sum_{i=1}^{n}{P}_{i}{Y}_{i}+2\sum_{i=1}^{n}{P}_{i}\times \left(1-{V}_{i}\right)-1$$

In the formula, P_i_ represents the proportion of the population or geographical area of the i-th province at the end of the year to the total population or geographical area of the country. Y_i_ represents the proportion of the quantity of OPCRs (the number of older people care institutions, the number of beds, the number of older people care service personnel, and the financial allocation received by the older people care industry) in the i-th province to the total quantity of OPCRs in the country. Vi represents the cumulative percentage of Y_i_ from i = 1 to i when ranking the per capita or per square kilometer OPCRs from low to high. The range of the Gini coefficient is (0, 1). Generally, G < 0.2 indicates absolute equity; 0.2–0.3 indicates equity; 0.3–0.4 indicates relatively equity; 0.4–0.5 indicates relatively inequity; G > 0.5 indicates extreme inequity [21, 10].

##### Theil’s index (T)

T proposed by the Dutch economist H. Theil, is used to explain the root of inequality [19, 21]. According to the decomposition principle of the Theil index, the smaller the value, the better the equity, and vice versa [21, 32]. The biggest advantage of using the Theil index to measure equity is that it can measure the contributions of intra-group and inter-group differences to the total difference. It has a certain complementarity with the Gini coefficient and its range is (0,1). Generally, the closer T is to 0, the better the equity, and vice versa. Its formula of calculation is:

$$T=\sum_{i=1}^{n}{P}_{i}{\text{log}}\frac{{P}_{i}}{{Y}_{i}}$$

Where P_i_ denotes the percentage of the population in each province of China and Y_i_ denotes the percentage of OPCR in each province of China. T can be decomposed into T_intra_ and T_inter_ [33]. The formula is as follows:

$$\begin{array}{l}T={T}_{{\text{int}}ra}+{T}_{{\text{int}}er}\\ {T}_{{\text{int}}ra}=\sum\limits_{g=1}^{k}{P}_{g}{T}_{g}\\ {T}_{{\text{int}}er}=\sum\limits_{g=1}^{k}{P}_{g}{\text{log}}\frac{{p}_{g}}{{Y}_{g}}\end{array}$$

Where T_g_ is the Theil index of three regional groups, including western, central, eastern, and northeastern regions, P_g_ is the ratio of the four regions’ population to China’s total population, and Yg is the ratio of the four regions’ OPCR to China’s total population. T_intra_ represents the intra-regional distribution of OPCR in the four regions. T_inter_ represents the interregional distribution of OPCR among the four regions. The intra-regional, inter-regional and different regional contribution rates can be obtained by the following equations.

$$\begin{array}{c}\mathrm{contribution \,rates \,of \,intraregion }={{\text{T}}}_{{\text{intra}}}/{\text{T}}\\ \mathrm{contribution \,rates \,of \,interregion }={{\text{T}}}_{{\text{inter}}}/{\text{T}}\\ \mathrm{contribution\, rates \,of \,the \,different \,regions }={{\text{P}}}_{{\text{g}}}{{\text{T}}}_{{\text{g}}}/{\text{T}}\end{array}$$

Old-care Resource Density Index (ORDI): shows the impact of population and geographical factors on the concentration of OPCRs. To avoid bias caused by using a single population or geographical dimension, its value is equal to the geometric mean of OPCRs per thousand people and OPCRs per square kilometer [33].

##### Old-care Resources Agglomeration Degree (ORAD)

The ORAD is introduced into the analysis of OPCRs based on China’s geographical characteristics and regional distribution. It is used to evaluate the equity and accessibility of OPCR allocation and aims to analyze the equity of the allocation of various types of OPCRs among provincial-level administrative units [34, 35]. Its concept and calculation formula are as follows:

$$OAR{D}_{{\text{i}}}=\frac{\left(O{R}_{i}/O{R}_{n}\right)\times 100\%}{\left({A}_{i}/{A}_{n}\right)\times 100\%}=\frac{O{R}_{i}/{A}_{i}}{O{R}_{n}/{A}_{n}}$$

OARD_i_ indicates the concentration of OPCR in a province, which is an indicator that reflects the proportion of total public OPCR that is concentrated in 1% of the geographical area of a province. OR_i_ is the number of public OPCRs of a given type in a province i, and OR_n_ is the total number of public OPCRs in the country. A_i_ is the geographical area of a province i, and A_n_ is the total geographical area of the country.

$$PA{D}_{i}=\frac{\left({P}_{i}/{P}_{n}\right)\times 100\%}{\left({A}_{i}/{A}_{n}\right)\times 100\%}=\frac{{P}_{i}/{A}_{i}}{{P}_{n}/{A}_{n}}$$

PAD_i_ denotes the population agglomeration of a province, reflecting the proportion of the national population that is concentrated in 1% of the geographical area of a province. Where P_i_ is the population of a given province i and P_n_ is the total national population. When evaluating the allocation of OPCR based on the concept of agglomeration degree, the criteria for assessing its equity are set as follows: If ORAD_i_ > 1, it means that the OPCRs in the region are fairly distributed geographically; if $\frac{{ORAD}_{{\text{i}}}}{{PAD}_{i}}$ tends to be close to 1, it means that the OPCRs in the region are just able to meet the older people care needs of the population in the region, $\frac{{ORAD}_{{\text{i}}}}{{PAD}_{i}}$ is greater than 1, which indicates that the OPCRs in the region are relatively excessive compared to the population collection, and $\frac{{ORAD}_{{\text{i}}}}{{PAD}_{i}}$ is less than 1, which suggests that the OPCRs in the region are insufficiently allocated to meet the local older people care needs [36, 37].

#### Dynamic DEA-SBM model

The traditional DEA models mainly include the Constant Returns to Scale (CCR) model based on scale invariance and the Variable Returns to Scale (BCC) model based on scale variability. Since both models are radial models, they cannot fully consider the issue of input-output flexibility. As a result, the efficiency values and ranking methods of the super-efficiency model are relatively inaccurate. Therefore, this paper uses the non-radial Slack-Based Measures (SBM) model to solve the above problems. At the same time, combining with the Dynamic DEA method proposed by Tone and Tsutsui [38], we explore the longitudinal changes of the efficiency of OPCR allocation over time. The Dynamic DEA model can make up for the insufficient consideration of inter-period efficiency and survival activities of the system in the traditional SBM-DEA model. By carrying over multiple consecutive periods, the dynamic changes of the efficiency of OPCR utilization in the time dimension are analyzed. In this study, since the input indicators of OPCRs are controllable, and the scale of the older people care industry is variable over time, a dynamic SBM-DEA model with input orientation and scale variability is used.

The basic model is as follows: with n DMUs passing through T terms, each DMU has inputs and outputs in period t and is connected to the next period t + 1 by Carry-over (See Additional file 1: Fig. S1).

Let n DMUs (j = 1, 2……n) pass through multiple T terms (t = 1, 2……T) and DMUs have m inputs (i = 1, k, m).

The input-oriented overall efficiency ${\theta }_{o}^{*}$ is defined by.

$${\theta }^{*}={\text{min}}\frac{1}{{\text{T}}}{\sum }_{t=1}^{T}{w}^{t}\left[1-\frac{1}{m+ngood}\left({\sum }_{i=1}^{m}\frac{{w}_{i}^{-}{s}_{it}^{-}}{{x}_{iot}}+{\sum }_{i=1}^{ngood}\frac{{s}_{it}^{good}}{{z}_{iot}^{good}}\right)\right]$$

subject to:

$$\begin{array}{c}{\sum }_{j=1}^{n}{z}_{ijt}^{\alpha }{\uplambda }_{{\text{j}}}^{{\text{t}}}={\sum }_{j=1}^{n}{z}_{ijt}^{\alpha }{\uplambda }_{{\text{j}}}^{{\text{t}}+1},\left(\forall i;t=1,K,T-1\right)\\ {z}_{iot}^{good}={\sum }_{j=1}^{n}{z}_{ijt}^{good}{\uplambda }_{{\text{j}}}^{{\text{t}}}-{s}_{it}^{good},\left(i=1,K,ngood;t=1,K,T\right)\\ {\sum }_{j=1}^{n}{\uplambda }_{{\text{j}}}^{{\text{t}}}=1,\left({\text{t}}=1,{\text{K}},{\text{T}}\right)\end{array}$$

where ${w}^{t}$ and ${w}_{i}^{-}$ are weights to term *t* and input *i* which are exogenously supplied according to their importance and satisfy the conditions as

$${\sum }_{t=1}^{T}{w}^{t}=T \,{\text{and}}\, {\sum }_{i=1}^{m}{w}_{i}^{-}=m$$

We define the term efficiency ${\theta }_{ot}^{*}$ by

$${\theta }_{ot}^{*}=1-\frac{1}{m+ngood}\left({\sum }_{i=1}^{m}\frac{{w}_{i}^{-}{s}_{it}^{-}}{{x}_{iot}}+{\sum }_{i=1}^{ngood}\frac{{s}_{it}^{good}}{{z}_{iot}^{good}}\right),\left(t=1,K,T\right)$$

This term efficiency expresses the input-oriented efficiency score for the term* t*. The overall efficiency during the period (${\theta }_{o}^{*}$) is the weighted average of the term efficiencies ${\theta }_{ot}^{*}$ as demonstrated below.

$${\theta }_{o}^{*}=\frac{1}{T}{\sum }_{t=1}^{T}{w}^{t}{\theta }_{ot}^{*}$$

#### A matching approach to efficiency and equity

**Fig. 1:**
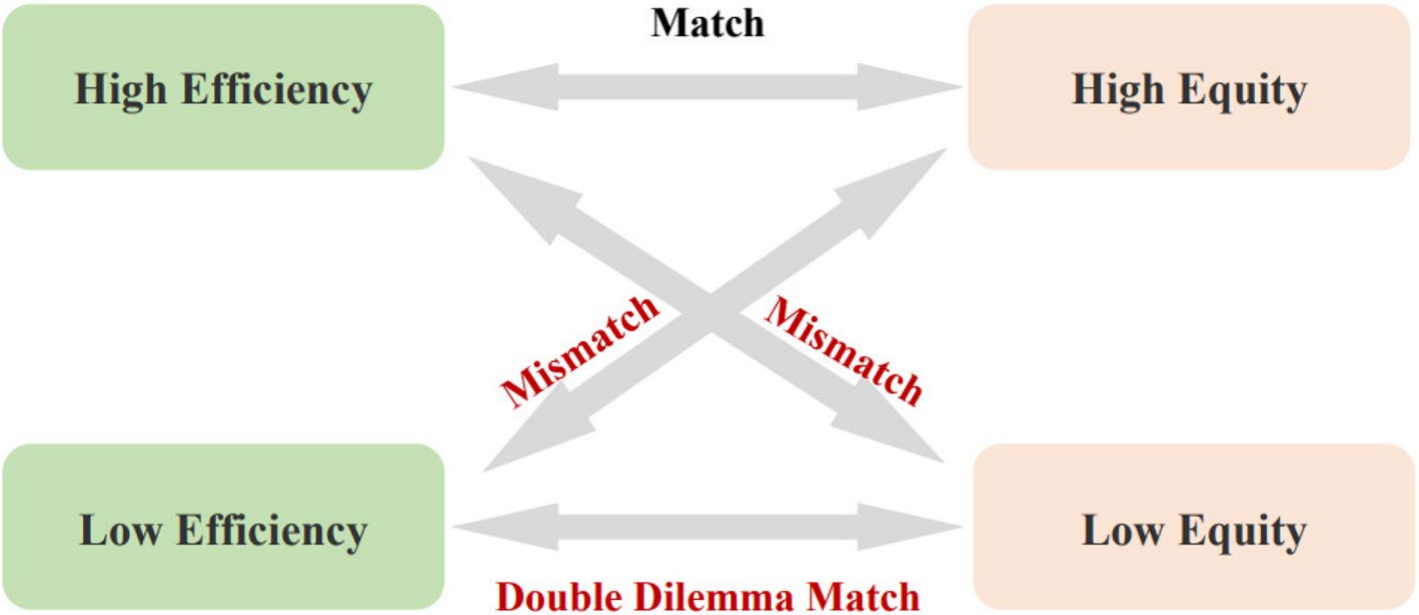
Coordination measurement of OPCRs’ equity and efficiency

This study plans to further explore the coordination between the results of Dynamic DEA and agglomeration degree by using ArcGIS software. Specifically, in terms of equity, we normalize the agglomeration degree result $\frac{{ORAD}_{{\text{i}}}}{{PAD}_{i}}$ using the Z-score method (the Z-score normalization method can standardize data into a set of values from less than zero to greater than zero, which is conducive to improving the convenience of processing data and calculation accuracy). In this study, we define that when the normalized value of $\frac{{ORAD}_{{\text{i}}}}{{PAD}_{i}}$ is positive (greater than zero), the equity of OPCRs in that region is higher, and it can meet the local older people care needs. When the normalized value is negative (less than zero), the equity of OPCRs in that region is lower, and it cannot meet the local older people care needs. In terms of resource allocation efficiency, we define regions where the efficiency is on the frontier (with an efficiency value of 1) as high, and the rest as low. We match them according to the method shown in Fig. 1. Finally, by plotting the matching results, we analyze the dynamic evolution of resource allocation equity and efficiency from 2009 to 2020.

#### Spatial autocorrelation

Spatial Autocorrelation is commonly used to explore whether there is statistical correlation between a certain variable or data in space, or to explore the potential mutual influence among several data indicators. The research theory inherits the first law of geography proposed by Swiss geographer Tobler, which states that everything is related to everything else, but near things are more related. As spatial autocorrelation can discover the distribution status and regular features of data in space, such as exploring the aggregation and dispersion states of data distribution, exploring the hot and cold spots of data distribution, it is often used to study the distribution of related indicators of public service facilities. In this study, the Global Moran’s I and Getis-Ord General G indices and the local indicator of spatial autocorrelation (LISA) and Getis-Ord Gi* tool were used to analyze the spatial distribution characteristics of the OPCR in each province, and to identify the aggregation and dispersion regions of data distribution.

##### Global spatial autocorrelation

Global Moran’s I reflect the overall spatial autocorrelation of the study area and is used to determine whether there is spatial autocorrelation in the research object as a whole. It is a spatial autocorrelation statistic for the entire study area [39].

The Global Moran’s I method of global spatial autocorrelation is given as

$$I=\frac{n}{{S}_{0}}\times \frac{{\sum }_{i=1}^{n}{\sum }_{j=1}^{n}{w}_{ij}\left({y}_{i}-\overline{y }\right)\left({y}_{j}-\overline{y }\right)}{{\sum }_{i=1}^{n}{\left({y}_{i}-\overline{y }\right)}^{2}},{S}_{0}=\sum_{i=1}^{n}\sum_{j=1}^{n}{w}_{ij}$$

##### Local spatial autocorrelation

The global autocorrelation statistic indicates the presence of clustering, while the local autocorrelation indicates the location and type of spatial correlation. In order to further study the distribution pattern of the OPCR, the local autocorrelation analysis method was used to identify the reachable local clusters. Due to the heterogeneity of space, there will be different clustering states in different geographical locations. LISA is suitable for studying the heterogeneity characteristics of the aggregation of OPCR [40].

The Getis-Ord Gi* tool is applicable for hotspot analysis, which can analyze the distribution of cold spots and hotspots of the PCMC.

Its calculation expression is as follows:

The LISA method of local spatial autocorrelation is given as

$${I}_{i}=\frac{n\left({x}_{i}-\overline{x }\right){\sum }_{i=1}^{n}{\sum }_{j=1}^{n}{w}_{i,j}\left({x}_{i}-\overline{x }\right)}{{\sum }_{i=1}^{n}{\left({x}_{i}-\overline{x }\right)}^{2}},$$

The Getis-Ord Gi* method of local spatial autocorrelation is given as

$${G}_{i}^{*}=\frac{{\sum }_{j=1}^{n}{W}_{i,j}{x}_{j}-\overline{X}{\sum }_{j=1}^{n}{w}_{i,j}}{S\sqrt[2]{\frac{\left[n{\sum }_{j=1}^{n}{w}_{i}^{2}-{\left({\sum }_{j=1}^{n}{W}_{i,j}\right)}^{2}\right]}{n-1}}},$$

where and are attribute values for features i and j; is the spatial weight between feature i and feature j; and n is the number of features in the dataset. When the statistic is higher than the mathematical expectation and passes the hypothesis test, it is a hot spot; otherwise, it is a cold spot [41].

## Results

### Current situation of OPCR allocation in China

From the China’s OPCR Allocation Status Change Chart (Additional file 1: Fig. S2), it can be seen that from 2009 to 2020, except for the basic stability of the number of older people care institutions, the number of beds, active employees, and government financial allocations have all increased. The number of active employees, beds, and government financial allocation per thousand people per square kilometer also showed a growth trend, especially the government financial allocation increased the fastest. The specific data are shown in Table 1.

**Table 1:** OPCR allocation in China from 2009 to 2020

Year	older people care institution			Active employees			Beds			Government financial allocation (ten thousand)		
	/1000 persons	/km2	Total	/1000 persons	/km2	Total	/1000 persons	/km2	Total	/1000 persons	/km2	Total
2009	0.0297	0.0041	39,671	0.1933	0.0268	257,940	2.1654	0.2998	2,889,793	0.0838	0.0116	111,824.5
2010	0.0298	0.0041	39,904	0.2041	0.0284	273,659	2.3480	0.3267	3,148,515	0.1235	0.0172	165,619.7
2011	0.0303	0.0042	40,868	0.2175	0.0304	293,409	2.5362	0.3550	3,421,682	0.3379	0.0473	455,837.7
2012	0.0326	0.0046	44,304	0.2434	0.0343	330,786	2.9191	0.4116	3,967,668	0.3715	0.0524	504,974.7
2013	0.0311	0.0044	42,474	0.2603	0.0369	355,921	3.1414	0.4456	4,295,111	0.7863	0.1115	1,075,034.6
2014	0.0240	0.0034	33,043	0.2423	0.0346	333,450	2.8350	0.4049	3,902,235	1.1019	0.1574	1,516,718.6
2015	0.0201	0.0029	27,752	0.2301	0.0330	318,240	2.5891	0.3716	3,581,382	1.3934	0.2000	1,927,421.1
2016	0.0205	0.0030	28,592	0.2433	0.0352	338,793	2.7205	0.3930	3,787,751	0.2288	0.0331	318,622.9
2017	0.0205	0.0030	28,770	0.2635	0.0383	368,946	2.7387	0.3978	3,834,502	2.0991	0.3049	2,938,929.3
2018	0.0204	0.0030	28,671	0.2674	0.0390	375,857	2.6994	0.3936	3,793,724	2.6332	0.3840	3,700,700.2
2019	0.0244	0.0036	34,369	0.3205	0.0469	451,979	3.1118	0.4552	4,387,879	3.2125	0.4700	4,529,919.6
2020	0.0270	0.0040	38,158	0.3670	0.0538	518,185	3.4575	0.5065	4,882,366	2.7313	0.4002	3,856,993.5

### The equity of OPCR allocation in China

This study starts from the perspective of population distribution and geographical area, and uses the Lorenz curve and Gini coefficient to describe the basic situation of OPCRs in China from 2013 to 2021. We calculate the Density Index and Aggregation to measure the equity of OPCR allocation.

### Gini coefficient and Lorenz curve diagram

**Fig. 2:**
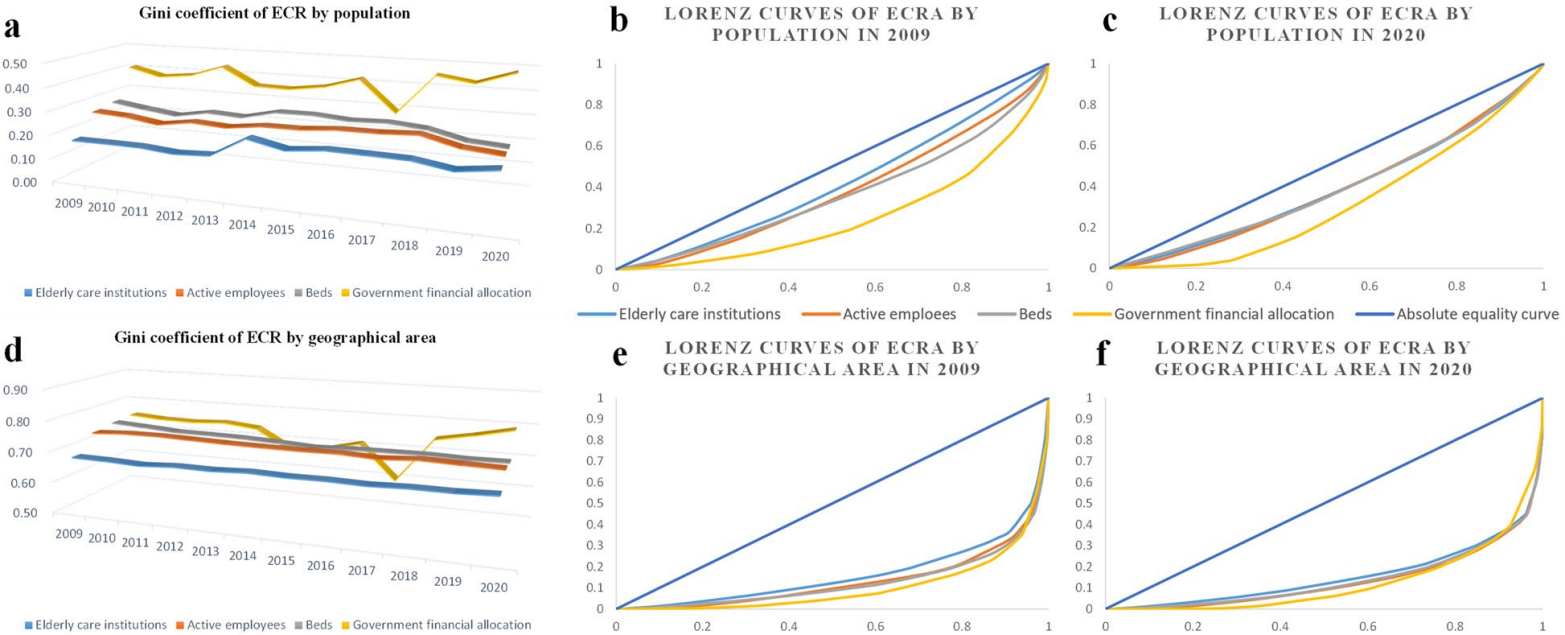
The equity of older people care resource allocation in China from 2009 to 2020. **a** and **d** show the G of older people care resources allocated by population and geographical area, respectively. **b** and **c** show the Lorenz curves of older people care resources allocated by population in 2009 and 2020, respectively. **e** and **f** show the Lorenz curves of older people care resources allocated by geographical area in 2009 and 2020, respectively

According to the Gini coefficient calculated from the perspective of population distribution (see Fig. 2), the distribution equity of older people care institutions is the highest, but there has been a slight decline over time; the distribution equity of active employees and beds is relatively poor, but it has been in a relatively reasonable range (0.2 ~ 0.3) for the past 12 years; the equity of government financial allocation is the worst, and its degree of inequity has continued to increase over time. The evaluation results of the Gini coefficient based on geographic latitude show that the Gini coefficients of older people care institutions, active employees, and beds have not changed much in the past 12 years, with values all greater than 0.6, indicating that their distribution is extremely unfair; the Gini coefficient of government financial allocation varies greatly, showing a downward trend from 09 to 17 years, reaching a minimum point of 0.59 in 17, and then rapidly increasing to 0.79 in 2020. The specific data are shown in Additional file 1: Tables S1 and S2.

Due to space limitations, this study only shows the Lorenz curves for 2009 and 2020 for reference. On the one hand, compared with the Lorenz curve based on geographic distribution, the Lorenz curves based on population distribution are significantly closer to the absolute equality line, which means that the distribution of OPCRs based on population allocation is fairer than based on geographic allocation. On the other hand, in the Lorenz curves based on population distribution and geographic distribution, the curve of government financial allocation is far from the absolute equality line in all other years except for the 2009 and 2020 curves. On the other hand, the 2020 curve is closer to the absolute equality line than the 2009 curve, indicating that the distribution equity of OPCRs has been optimized over time. See Fig. 2 for details.

### Theil index

**Fig. 3:**
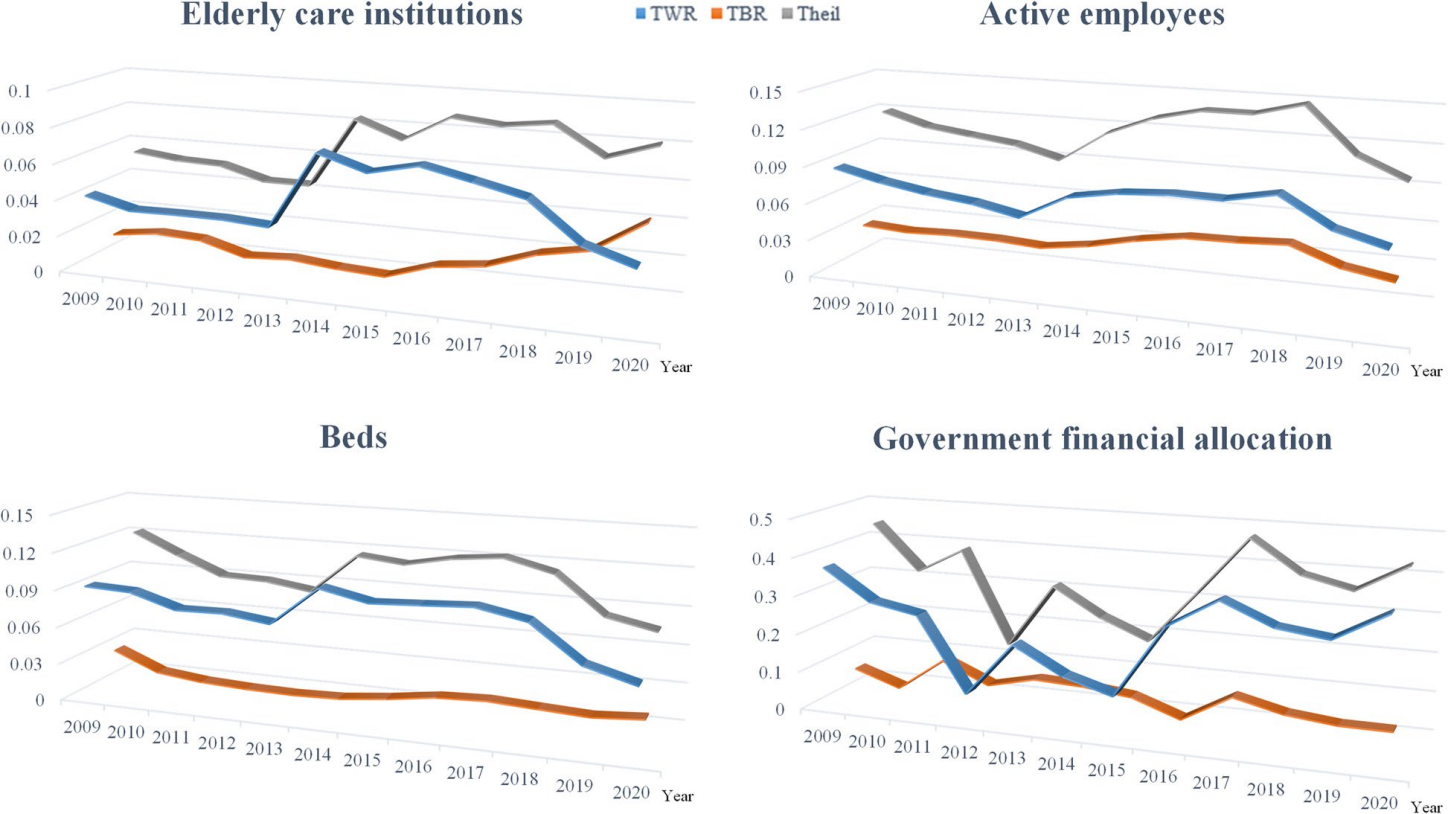
Theil Index of OPCR allocation in China from 2009–2020

**Fig. 4:**
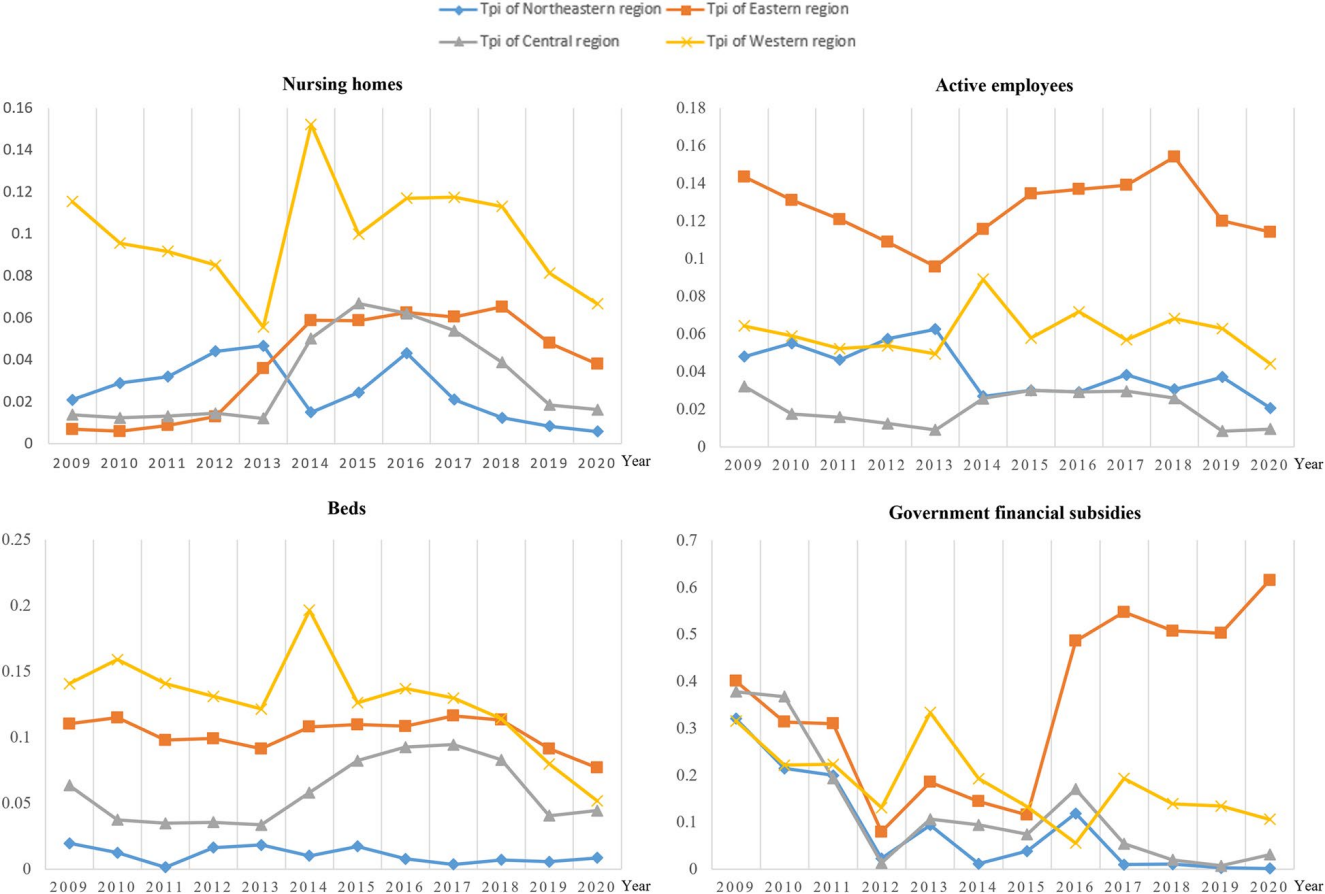
Theil Index of OPCR allocation among different regions from 2009 to 2020

The results of using the Theil index to calculate the equity of OPCR allocation are basically consistent with the results of the Gini coefficient and Lorenz curve (see Figs. 3 and 4 for details). Through in-depth analysis of the sources of inequality, it can be found that the difference in OPCR allocation between provinces within the region is significantly greater than that between regions: the regional differences in older people care institutions and on-the-job staff have a trend of narrowing year by year within the region, while the differences between regions gradually expand; the number of beds and government funding are relatively stable (see Additional file 1: Table S3 for details). Continuing to decompose the differences within the region, we can find that, first, in the past 12 years, the difference in older people care institution allocation has gradually narrowed in the western region, expanded year by year in the eastern region, and first expanded and then narrowed in the central and northeastern regions. Currently, the pattern is western > eastern > central > northeastern. Second, in the past 12 years, the difference in on-the-job staff allocation has been relatively stable, and currently shows the pattern of eastern > western > central > northeastern. Third, the number of older people care beds shows a similar trend to that of older people care institutions, and currently shows the pattern of eastern > western > central > northeastern. Finally, the difference in government funding allocation gradually narrowed in the northeastern, central, and western regions from 2009 to 2020, while it gradually expanded in the eastern region. Currently, it also shows the pattern of eastern > western > central > northeastern (see Additional file 1: Table S4 for details). To clarify the equity of OPCR allocation in the four regions, the Theil index for each region is calculated separately, and it can be seen that older people care institutions and on-the-job staff are most fairly distributed in the central region, while the distribution is the most unfair in the western region; the number of beds and government funding are most fairly distributed in the northeastern region, while the distribution is the most unfair in the eastern region (See Additional file 1: Table S5 for details).

### Resource density index and aggregation

**Fig. 5:**
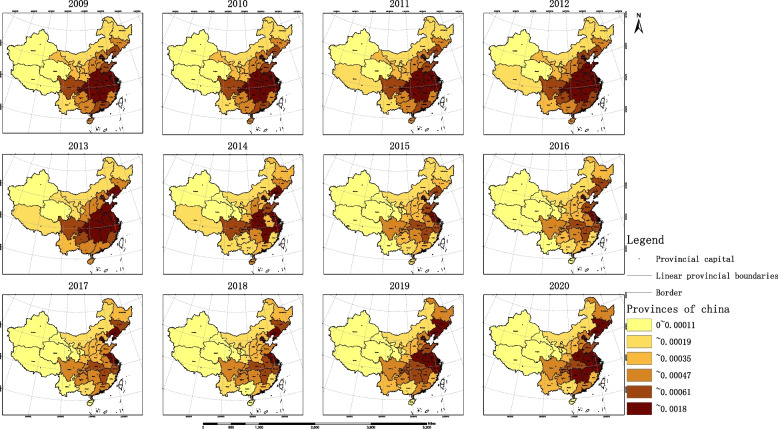
ORDI of older people care institutions

The OPCR density index is an equity indicator that takes into account both population and geographical factors to measure the concentration of OPCRs. The results show that the centers of concentration of older people care institutions, on-the-job staff, beds, and financial allocations gradually moved from the central region to the eastern coastal region from 2009 to 2020 (see Fig. 5 and Additional file 1: Figs. S3-S5 for details). Among them, the center of gravity of government financial allocation has always been located in the eastern coastal region of China, but before 2016, it had a tendency to tilt towards the western region and gradually disappeared afterwards.

**Fig. 6:**
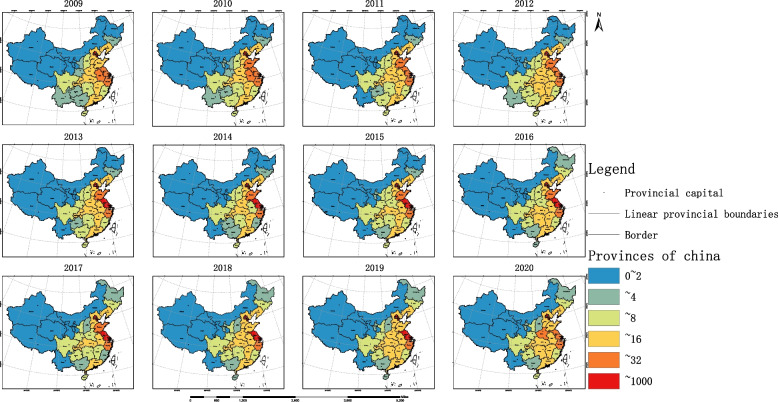
HRAD results in China by province

By calculating the ORAD values of each province, the results are shown in Fig. 6: from 2009 to 2020, China’s main OPCRs were concentrated in the eastern region, especially in the provinces of Beijing, Tianjin, and Shanghai, which have had an overflow of OPCRs for many years, resulting in their OPCR accessibility always being at the highest level in the country. However, the accessibility of OPCRs in most western regions, as well as some central and northeastern regions, is poor and cannot meet the needs of the population in these areas.

### The efficiency of OPCR allocation in China

The statistical description results of efficiency analysis data are shown in Additional file 1: Table S6. In the input variables, the number of older people care institutions rapidly decreased after 2013 and gradually rebounded after 2018. The number of staff at the end of the year and the number of beds showed a decreasing trend in 2013–2014 and an increasing trend in other years. The total value of fixed assets and the total area of older people care institutions showed an overall increasing trend but experienced a rapid decline in 2016–2017 followed by a rapid increase after 2017. In the output variables, the number of older people care institution residents, total days, self-care service users, and assisted service users showed an increasing trend before 2013 and a decreasing trend after 2013. The number of self-care, assisted care, and nursing service users, as well as the number of rehabilitation and medical outpatient visits, all reached their lowest point in 2017.

According to Table 2, the overall average efficiency of the allocation of OPCRs in China from 2009 to 2020 showed a low point in 2016 at 0.8664 and a high point in 2020 at 0.9396. The overall efficiency showed a pattern of increasing–decreasing-increasing, with turning points in 2012 and 2016.

**Table 2:** Efficiency values of OPCR allocation in China

	Overall	2009	2010	2011	2012	2013	2014	2015	2016	2017	2018	2019	2020
Avg	0.9095	0.8607	0.9068	0.9311	0.8851	0.9238	0.9208	0.8968	0.8664	0.9057	0.9027	0.9205	0.9396
Max	1	1	1	1	1	1	1	1	1	1	1	1	1
Min	0.6133	-0.9428	0.6064	0.5635	0.4959	0.6223	0.5912	0.5624	0.5299	0.5022	0.635	0.6239	0.6214
SD	0.1054	0.36	0.1448	0.1433	0.1619	0.1229	0.1341	0.1399	0.1648	0.1632	0.1319	0.1343	0.1238

Sorting the average efficiency of OPCR allocation among the 31 provincial-level administrative regions from 2009 to 2020, the provinces that consistently maintained their position in the top tier of efficiency for the 12 years included Tianjin, Heilongjiang, Shanghai, Jiangsu, Guangdong, Sichuan, and Tibet. The five provinces with the lowest rankings were Yunnan, Hebei, Guizhou, Fujian, Gansu, and Shaanxi, with Shaanxi having the lowest efficiency value of 0.6133. However, there has been a gradual improvement trend since 2017 (See Additional file 1: Table S7 for details).

### Analysis of the coordination between the equity and efficiency of OPCR allocation

To further illustrate the balance between equity and efficiency in the allocation of OPCRs, this study matched the indicators of resource equity satisfaction and utilization efficiency for each provincial-level administrative region and plotted the results as follows:

In 2009, 32.3% of provinces achieved high equity and efficiency in the allocation of OPCRs, scattered throughout the western, central, and eastern regions. 35.3% of regions had high resource utilization rates, but OPCRs were insufficient to meet local needs, mostly located in high latitude areas in northern China. Two provinces had high resource equity and low allocation efficiency, namely Anhui and Chongqing. 25.8% of provinces faced the dual challenges of low efficiency and low equity, mainly in western and central regions.

**Fig. 7:**
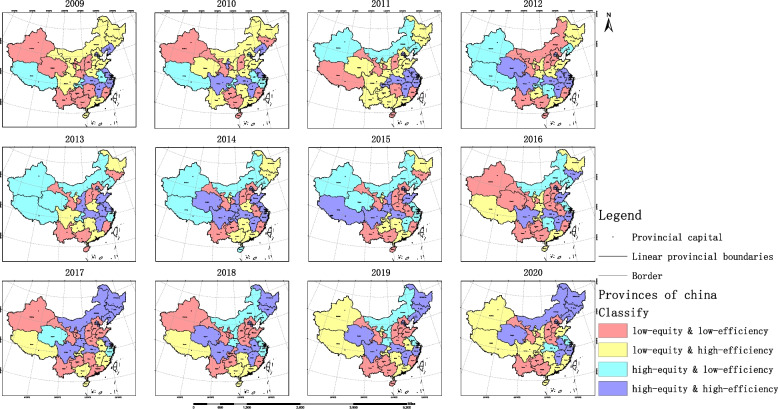
Coordination between the equity and efficiency

From 2009 to 2020, China’s older people care industry has made significant progress, especially in the eastern and northeastern regions. By 2020, 41.9% of provinces had high efficiency and high equity in the allocation of OPCRs, mainly concentrated in the northeastern region and the eastern coastal areas. Only Hubei Province had high resource equity but low efficiency. 35.5% of provinces had high efficiency but low equity, mostly concentrated in western regions. 19.4% of provinces faced dual challenges, namely Hebei, Shanxi, Guangxi, Guizhou, Yunnan, and Gansu (See Fig. 7, Additional file 1: Table S8 for details).

Global Moran’s I results showed that the accessibility of OPCRs in China exhibited spatial clustering within the 90% confidence interval from 2009 to 2015 and 2018 to 2020. This indicates that provinces with similar levels of equity tend to spatially cluster together, while provinces with different levels of equity are spatially separated. However, there was no spatial clustering of equity in the overall allocation of resources in 2016 and 2017. Among these years, the distribution was least coordinated in 2013 (Moran’s I = 0.082967, z-score = 1.95596), while the coordination improved in 2020, with the weakest positive correlation (Moran’s I = 0.012046, z-score = 1.686258). Notably, there was a significant improvement in the coordination of equity in the allocation of OPCRs after 2016, as shown in Table 3. Furthermore, there was no significant spatial clustering of efficiency in the allocation of OPCRs across provinces in China, as indicated in Table 3.

**Table 3:** Global spatial autocorrelation analysis results of equity and efficiency

Year	Equity					Efficiency				
	Moran’s I	Expected Index	Variance	z-score	*p*-value	Moran’s I	Expected Index	Variance	z-score	*p*-value
2009	0.064729	-0.033333	0.002694	1.88926	0.058857	-0.020113	-0.030303	0.00211	0.221848	0.824432
2010	0.04477	-0.033333	0.002099	1.704959	0.088202	0.002121	-0.030303	0.007596	0.372038	0.709865
2011	0.064261	-0.033333	0.003297	1.699743	0.089179	-0.087761	-0.030303	0.00683	-0.695222	0.486916
2012	0.067332	-0.033333	0.0022	2.146202	0.031857	-0.090175	-0.030303	0.007262	-0.702594	0.482309
2013	0.082967	-0.033333	0.003535	1.95596	0.05047	-0.062368	-0.030303	0.007001	-0.383205	0.701568
2014	0.073259	-0.033333	0.003108	1.912021	0.055873	-0.064216	-0.030303	0.007077	-0.403114	0.686865
2015	0.063432	-0.033333	0.002927	1.788541	0.073689	-0.049835	-0.030303	0.007253	-0.229346	0.8186
2016	0.055605	-0.033333	0.004355	1.347685	0.17776	-0.094218	-0.030303	0.00746	-0.740027	0.459284
2017	0.011411	-0.033333	0.000903	1.488755	0.136552	0.094662	-0.030303	0.007029	1.490503	0.136092
2018	0.013079	-0.033333	0.0007	1.754462	0.079351	-0.010962	-0.030303	0.007349	0.225604	0.821509
2019	0.015186	-0.033333	0.000648	1.906069	0.056641	0.050324	-0.030303	0.007186	0.951132	0.341537
2020	0.012046	-0.033333	0.000724	1.686258	0.091746	0.09991	-0.030303	0.006817	1.577117	0.114769

**Fig. 8:**
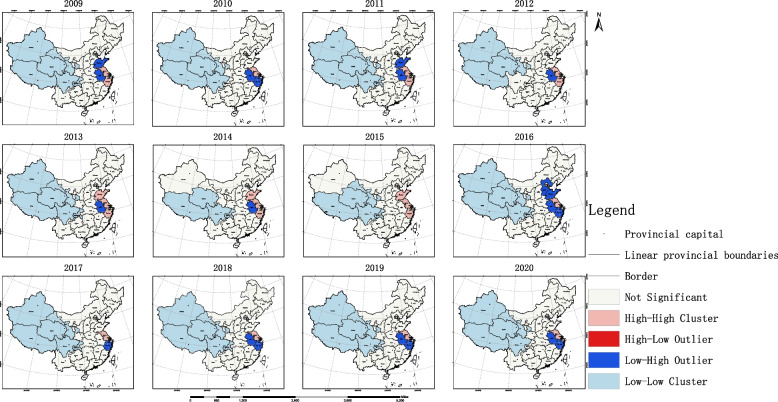
Local spatial autocorrelation analysis results of OPCR equity

**Fig. 9:**
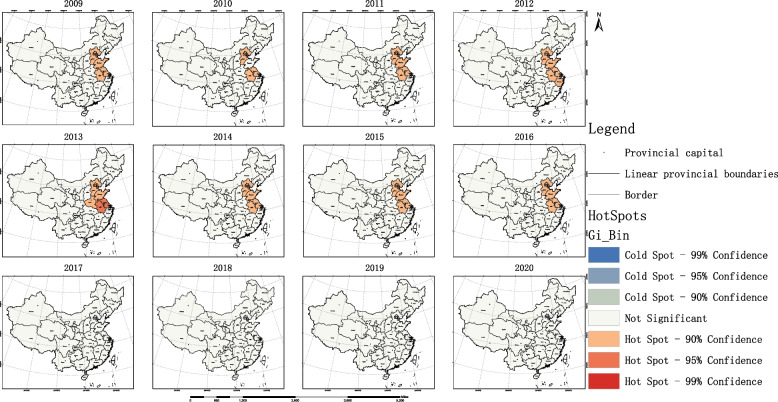
Hot and cold spot analysis results of OPCR equity

Based on the results of the global spatial autocorrelation analysis, this study employed local spatial autocorrelation analysis to identify specific discrete and clustered regions. The results are as follows: The LISA map revealed high-high clustering areas of accessibility to OPCRs in the southeastern coastal provinces around Shanghai, while low-low clustering areas were primarily observed in western provinces of China. Taking the year 2020 as an example, Shanghai was the only high-low region, Jiangsu was a high-high region, Anhui and Zhejiang were low-high regions, and Xinjiang, Tibet, Qinghai, Gansu, and Sichuan were low-low regions. Please refer to Fig. 8 for details. The hotspot analysis showed that prior to 2016, there were multiple hotspot provinces in terms of accessibility to OPCRs, mostly located in eastern China. Starting from 2017, Shanghai became the sole hotspot region. Please see Fig. 9 for reference.

**Fig. 10:**
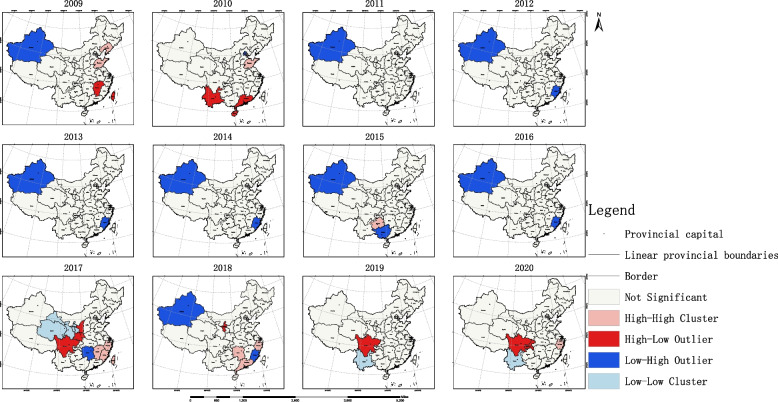
Local spatial autocorrelation analysis results of OPCR efficiency

**Fig. 11:**
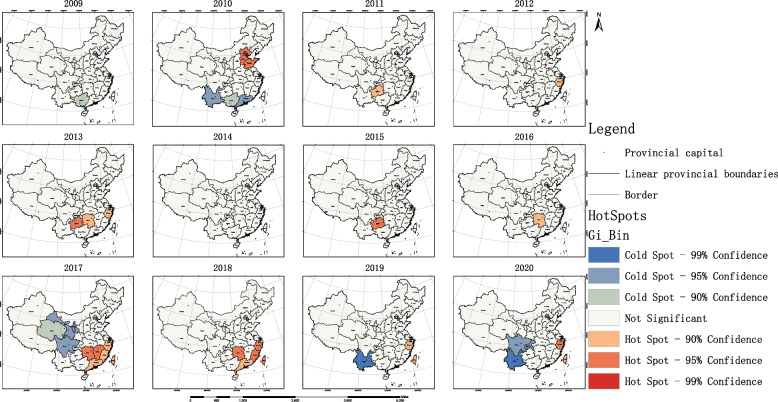
Hot and cold spot analysis results of OPCR efficiency

Regarding the coordination of efficiency in the allocation of OPCRs in China, from 2009 to 2020, Xinjiang appeared as a consistently low-efficiency province, but there was improvement starting from 2019. In 2020, Zhejiang was the high-high region, Sichuan and Chongqing were the high-low regions, and Yunnan was the only low-low region, with no low-high regions present anymore. Please see Fig. 10 for details. The hotspot analysis displayed a changing trend in the hotspot regions of efficiency in the allocation of OPCRs in China, progressing from Beijing and its surrounding areas to central regions such as Guizhou and finally to the southeastern coastal regions. The major coldspot regions shifted from central provinces like Yunnan to western provinces like Qinghai and then back to central provinces like Yunnan. By 2020, the main hotspot region appeared in Zhejiang province, while the primary coldspot region emerged in Yunnan province. Please refer to Fig. 11 for further information.

## Discussion

From 2009 to 2020, China’s older people care industry has experienced rapid development under the strong advocacy of the government. In terms of equity, the total amount of OPCRs has been significantly increased, but there are still significant differences in resource allocation between regions and provinces. The utilization efficiency of OPCRs has improved significantly, and by 2020, only a small number of central and western provinces were not at the forefront of efficiency.

Coordination analysis shows that the central and eastern regions exhibit a polarized pattern in balancing equity and efficiency, while the main problem in western regions is still the scarcity of OPCRs.

Over the past 12 years, the number of older people care workers, the number of beds, and the amount of government allocation in China have all shown an upward trend in terms of per thousand people and per square kilometer. In addition, the number of older people care institutions has slightly decreased, mainly due to government policies in recent years that have restricted the blind expansion of older people care institutions in terms of quantity and scale, and shifted the development direction of older people care institutions from quantity to quality.

According to the Gini coefficient and Lorenz curve, this study draws two conclusions. Firstly, the distribution of older people care institutions, bed numbers, and staff numbers per capita is relatively fair, while the equity of government financial allocation is poor. The main reason for this phenomenon may be that the amount of fiscal appropriations depends largely on the tax revenue of each city, which is deeply influenced by the local economic development level [42]. In addition, there are significant differences in economic development levels among the western, central, eastern, and northeastern regions of China. Therefore, the government is recommended to improve the redistribution system and increase financial support to underdeveloped areas, especially in the western regions.

Secondly, the equity of OPCR allocation according to population distribution is better than that according to geographical regions, which is consistent with other studies on OPCR allocation in China [43]. There are two possible explanations for this result: one is that some provinces with large geographical areas, such as Xinjiang, Inner Mongolia, and Tibet, have a lower level of economic development and face a shortage of OPCRs [5]; the other is that the Chinese government uses the number of older people receiving medical treatment as a reference when formulating relevant OPCR allocation standards, without considering factors such as geographical distribution and service coverage. The farther residents are from the OPCRs, the more time and economic costs they will incur, and the less willing they will be to receive older people care services. This is extremely unfavorable for China to alleviate the aging trend [44]. Therefore, when formulating OPCR allocation plans, the government needs to consider both population and geographical factors.

The Theil index results show that the un equity of OPCR allocation in China mainly comes from regional differences. Especially in the western region, there are significant differences in OPCR allocation among provinces, and their contribution to the national difference is also far greater than that of other regions, which is consistent with the research results of Li Fen et al. [45]. The ORDI results show that, at present, OPCRs are concentrated in the eastern coastal areas, and OPCRs in the western region are extremely scarce. The concentration analysis also indicates that the accessibility and demand satisfaction of OPCRs in the western region are the worst. In summary, it can be found that the development of older people care services in the western region of China is extremely unbalanced. Improving the equity of OPCR allocation in the western region can effectively enhance the equity of OPCR allocation in China. Therefore, the government is recommended to attach great importance to the equity of OPCR allocation in the western region, guarantee the older people care service needs of residents in underdeveloped provinces and remote cities such as Xinjiang, Tibet, Qinghai, and Inner Mongolia, and encourage high-level older people care service talents and relevant professionals with higher education backgrounds to flow reasonably from developed provinces to remote provinces under the guidance of policies and the support of central subsidy funds, so that residents in remote areas can obtain relatively high-quality OPCRs and services.

According to the efficiency analysis results, the average allocation efficiency of OPCRs in China is 0.9095. After 12 years of vigorous development, in 2020, 77.42% of the provinces’ resource allocation efficiency was at the forefront. Only a small number of provinces had unsatisfactory results in the allocation of OPCRs, which were all located in the central or western regions of China. This result is consistent with the research conducted by Liu Yiping and others [46].

This indicates the importance of establishing cooperation and communication mechanisms among various regions, and encouraging the backward provinces (Hebei, Shanxi, Hubei, Guangxi, Guizhou, Yunnan, and Gansu) to learn from the advanced concepts and successful practices of the eastern cities with high efficiency in OPCR utilization. The Chinese government has been promoting regional exchanges and cooperation, and currently, this policy has achieved certain results. It is believed that with the improvement of relevant systems and the implementation of specific measures, the development of China’s older people care industry will be further enhanced.

Regarding the analysis of coordination, in 2020, after 12 years of coordinated development, the three provinces in Northeast China were all evaluated as high-equity and high-efficiency. In the eastern region, five provinces were evaluated as high-equity and high-efficiency, while four provinces were evaluated as low-equity and high-efficiency, and Hebei Province faced the dual challenge of low-equity and low-efficiency. In the central region, four provinces were evaluated as high-equity and high-efficiency, while Hubei was evaluated as high-equity and low-efficiency, and Shanxi was evaluated as low-equity and low-efficiency. These results indicate that the central and eastern regions exhibit polarization in terms of the coordination of equity and efficiency. It reminds the governments at all levels to attach great importance to the integrated development within the region, strengthen regional older people care planning, focus on underdeveloped provinces, and promote coordinated development of OPCRs in the region.

In the western region, only Inner Mongolia was evaluated as high-equity and high-efficiency, while the rest of the provinces were evaluated as low-equity and high-efficiency or low-equity and low-efficiency. This indicates that the western provinces of China are currently facing the dilemma of insufficient OPCRs [47]. The government needs to strengthen cooperation and communication between the western region and other regions, promote the flow of OPCRs between regions, focus on improving the coverage and service quality of OPCRs in underdeveloped areas, and make efforts to develop the older people care industry. At the same time, the western provinces should learn from the management experience and specific measures of the high-efficiency provinces with sufficient OPCRs, prioritize efficiency improvement in the limited OPCR situation, so as to avoid wasting resources when obtaining corresponding OPCRs in the future, and strive to achieve the coordinated dynamic sustainable development of equity and efficiency in the allocation of OPCRs in China.

Further conduct spatial statistical analysis on OPCRs to clarify their spatial coordination. The findings from our study shed light on the spatial patterns and equity in the accessibility and allocation of OPCRs in China. The results of the Global Moran’s I analysis revealed spatial clustering of resource accessibility within the 90% confidence interval during the periods of 2009–2015 and 2018–2020. This indicates that provinces with similar levels of equity tend to cluster together spatially, while those with different levels of equity tend to be spatially separated. However, no significant spatial clustering of equity was observed in the overall resource allocation in 2016 and 2017.

It is worth noting that 2013 exhibited the least coordination in resource distribution, while 2020 showed improved coordination with the weakest positive correlation. This suggests that efforts have been made to enhance the equity in the allocation of OPCRs over the years, particularly after 2016. These findings highlight the progress made in promoting equity and equal access to OPCRs across provinces in China.

Moreover, the analysis of local spatial autocorrelation provided insights into specific regions of high-high and low-low clustering. The high-high clustering areas of resource accessibility were predominantly located in the southeastern coastal provinces surrounding Shanghai, while the low-low clustering areas were primarily observed in the western provinces. This information can serve as a valuable reference for policymakers and stakeholders in identifying regions that require targeted interventions to address disparities in OPCR availability.

Furthermore, the examination of resource allocation efficiency revealed interesting trends. Xinjiang consistently appeared as a low-efficiency province during most of the studied period, although improvements were observed starting from 2019. By 2020, Zhejiang emerged as the high-high region in terms of resource allocation efficiency, followed by Sichuan and Chongqing as the high-low regions. Yunnan was the only province classified as a low-low region, with no remaining low-high regions. These findings highlight the need for continued efforts to improve OPCR allocation efficiency, particularly in regions that lag behind.

The analysis of hotspot regions in resource accessibility and allocation efficiency provided additional insights. Prior to 2016, there were multiple hotspot provinces in terms of resource accessibility, mainly concentrated in eastern China. However, starting from 2017, Shanghai became the sole hotspot region, indicating its exemplary efforts in providing accessible OPCRs. In terms of resource allocation efficiency, the hotspot regions changed over time, shifting from Beijing and its surrounding areas to central regions such as Guizhou, and finally to the southeastern coastal regions. This demonstrates the evolving nature of resource allocation patterns and the need for adaptive policies and strategies.

Overall, our study highlights both the progress made and the ongoing challenges in achieving equitable access and efficient allocation of OPCRs in China. The findings contribute to the understanding of spatial patterns and can inform policymakers and stakeholders in formulating targeted interventions to improve resource accessibility and allocation efficiency across different regions.

## Limitations

Although in this study, we attempted to comprehensively evaluate the equity and efficiency of the allocation of OPCRs in China using various methods and dynamic DEA models, there are still some limitations. First, the selection of indicators is based on previous research. However, like most studies, due to the availability of data, the output indicators do not include quality indicators such as older people health recovery, satisfaction, and staff self-evaluation. Secondly, this study is based on a national perspective, and the analysis of the allocation of OPCRs in China is at a macro level. In the future, the research scale can be narrowed, and more precise accessibility evaluation methods can be applied to make a more detailed evaluation of the allocation of OPCRs and provide guidance suggestions.

## Conclusions

This study examined trends in the equity and efficiency of OPCR allocation in China from 2009 to 2020. The results revealed several noteworthy findings. Firstly, while the overall provision of OPCRs has improved substantially over the past decade as evidenced by rising beds, staffing and financial investments, significant disparities persist both within and across Chinese regions. The Gini coefficient and Theil index consistently showed the eastern region enjoys substantially higher OPCR accessibility compared to other areas. Spatially, OPCR allocation clusters around coastal and southeastern provinces. Secondly, the coordination between equity and efficiency has strengthened in recent years but remains imperfect. By 2020, only 42% of provinces achieved high levels of both indicators. A sizable minority still face dual challenges of low efficiency and inequitable distribution. These provinces are concentrated in western regions highlighting the need for targeted support. Thirdly, local spatial statistical analyses found Shanghai has emerged as the dominant hotspot of OPCR equity and efficiency. However, coldspots consistently include inland provinces like Yunnan highlighting pressing needs for strategic resource reallocation.

To sum up, according to the research results, in order to further improve the efficiency and equity of the allocation of OPCRs, there are three aspects that need to be compensated. Firstly, a balanced approach is needed that considers both expanding total resources as well as optimizing allocation schemes according to local conditions. Secondly, targeted support should focus on increasing inputs while promoting efficiency in provinces facing dual issues. Lastly, redistribution mechanisms must be strengthened to narrow inter-regional disparities over time. Based on this, to address the uneven allocation of OPCRs across China and promote a more rational, coordinated system, this study puts forth the following targeted recommendations:

### Policy recommendations

Establish a central regulatory body to oversee strategic resource planning, allocation standards, redistribution schemes, performance evaluation, and multi-stakeholder coordination at national and sub-national levels. Allocation standards should incorporate demographic as well as geographic factors through a needs-based funding formula directing higher investments to districts with rapidly aging populations but scarce resource infrastructure, based on objective criteria like dependency ratios and population densities.

Strengthen regional resource pooling and cooperative arrangements between more developed eastern provinces and inland counterparts through incentives to foster balanced development. Support targeted training programs, staff exchange initiatives, and communities-of-practice across provinces to facilitate mobility of resources and sharing of best practices, with the aim of lifting skills and productivity in lower-efficiency settings.

Leverage public-private partnerships to co-fund infrastructure development projects expediting construction of nursing homes, day care facilities, and telehealth networks in resource-poor regions. Also support community-based initiatives like village cooperatives, volunteer caregiver networks and home-help services to meet local older people care needs through tailored solutions.

Implement outcome-based performance evaluation of provinces using indicators covering both resource availability and utilization. Underperforming provinces could receive performance-based support while higher-capacity regions may face reallocation incentives.

Launch talent incentive programs to attract and retain skilled geriatric healthcare workers in underserved remote/rural communities through targeted bonuses, subsidy grants and educational sponsorship.

By embracing these evidence-based, multi-pronged reforms, Chinese authorities can optimize resource use, address social inequities and build a sustainable, people-centered older people care support system benefiting all citizens as the population increasingly ages across its vast territory. Ongoing review and refinement will ensure the evolving needs of an aging population are met.

### Supplementary Information

**Additional file 1: Figure S1.** Basic Mechanism of the Dynamic DEA Model. **Figure S2.** Total OPCR in China from 2009 to 2020. **Table S1.** Gini coefficient of OPCR by population. **Table S2.** Gini coefficient of OPCR by geographical area. **Table S3.** Theil Index of OPCR allocation in China from 2009-2020. **Table S4.** Contribution to the overall differences between regions from 2009 to 2020. **Table S5.** Theil Index of OPCR allocation among different regions from 2009 to 2020. **Figure S3.** ORDI of Active employees. **Figure S4.** ORDI of Beds. **Figure S5.** ORDI of Government financial allocation. **Table S6.** Descriptive statistics for inputs and outputs in China. **Table S7.** Efficiency values of OPCR allocation in China by provincial-level. **Table S8.** Statistical description of Balance evaluation between efficiency and equity.

## Data Availability

The data for this study were obtained from publicly available government statistical yearbooks. The data can be made available upon reasonable request from the Corresponding author.
