# Supplementary material for: Bridging the gap: coordinating equity and efficiency in older people care resource allocation in China
Source: BMC Geriatr. 2024 Feb 16;24:165. doi: 10.1186/s12877-024-04696-w (PMC10874015; doi:10.1186/s12877-024-04696-w)
Supplement: Supplementary file 1 — Additional file 1: Figure S1. Basic Mechanism of the Dynamic DEA Model. Figure S2. Total OPCR in China from 2009 to 2020. Table S1. Gini coefficient of OPCR by population. Table S2. Gini coefficient of OPCR by geographical area. Table S3. Theil Index of OPCR allocation in China from 2009-2020. Table S4. Contribution to the overall differences between regions from 2009 to 2020. Table S5. Theil Index of OPCR allocation among different regions from 2009 to 2020. Figure S3. ORDI of Active employees. Figure S4. ORDI of Beds. Figure S5. ORDI of Government financial allocation. Table S6. Descriptive statistics for inputs and outputs in China. Table S7. Efficiency values of OPCR allocation in China by provincial-level. Table S8. Statistical description of Balance evaluation between efficiency and equity. [file 12877_2024_4696_MOESM1_ESM.docx]

**Supplementary Information**

Additional file 1 (.docx) provides statistical descriptions and specific results of the equity, efficiency, and coordination analyses, as well as other additional information.

Figure S1 Basic Mechanism of the Dynamic DEA Model；

Figure S2 Total OPCR in China from 2009 to 2020；

Table S1 Gini coefficient of OPCR by population；

Table S2 Gini coefficient of OPCR by geographical area；

Table S3 Theil Index of OPCR allocation in China from 2009-2020；

Table S4 Contribution to the overall differences between regions from 2009 to 2020；

Table S5 Theil Index of OPCR allocation among different regions from 2009 to 2020；

Figure S3 ORDI of Active employees；

Figure S4 ORDI of Beds；

Figure S5 ORDI of Government financial allocation；

Table S6 Descriptive statistics for inputs and outputs in China；

Table S7 Efficiency values of OPCR allocation in China by provincial-level；

Table S8 Statistical description of Balance evaluation between efficiency and equity；


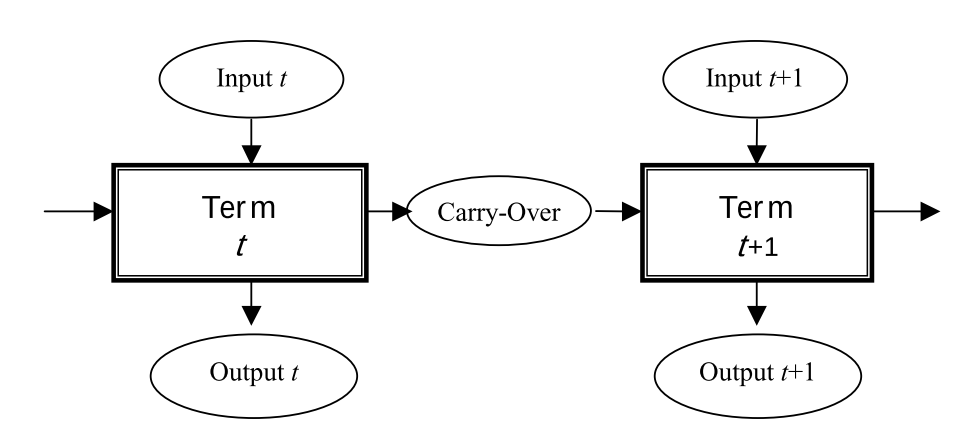


Figure S1 Basic Mechanism of the Dynamic DEA Model


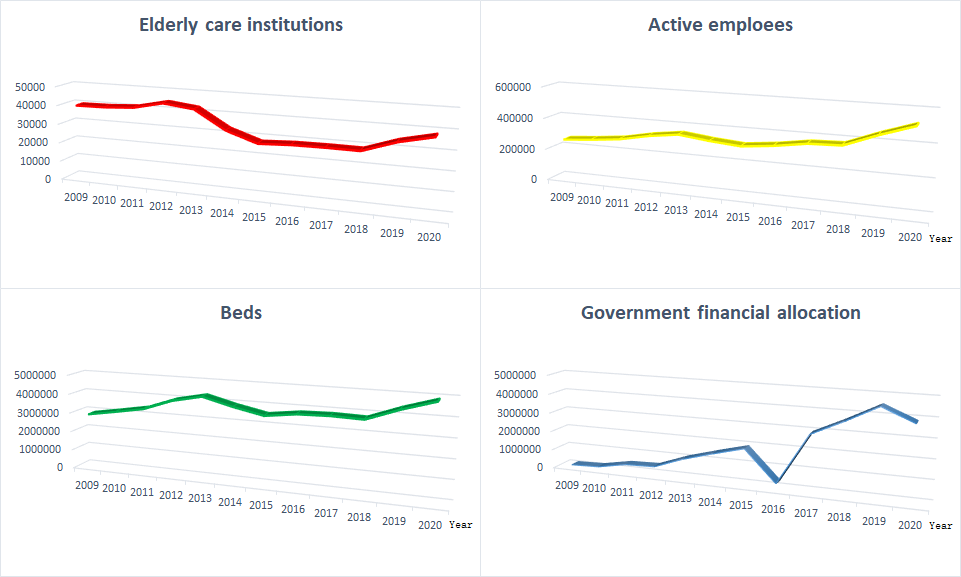


Figure S2 Total ECR in China from 2009 to 2020

| Table S1 Gini coefficient of ECR by population | | | | | |
| --- | --- | --- | --- | --- | --- |
| Year | Elderly care institutions | | Active employees | Beds | Government financial allocation |
| 2009 | 1.74E-01 | 2.61E-01 | | 2.73E-01 | 4.07E-01 |
| 2010 | 1.74E-01 | 2.53E-01 | | 2.52E-01 | 3.73E-01 |
| 2011 | 1.73E-01 | 2.32E-01 | | 2.35E-01 | 3.88E-01 |
| 2012 | 1.61E-01 | 2.53E-01 | | 2.60E-01 | 4.35E-01 |
| 2013 | 1.68E-01 | 2.44E-01 | | 2.53E-01 | 3.59E-01 |
| 2014 | 2.51E-01 | 2.63E-01 | | 2.86E-01 | 3.56E-01 |
| 2015 | 2.20E-01 | 2.63E-01 | | 2.87E-01 | 3.76E-01 |
| 2016 | 2.35E-01 | 2.76E-01 | | 2.75E-01 | 4.19E-01 |
| 2017 | 2.32E-01 | 2.76E-01 | | 2.80E-01 | 2.87E-01 |
| 2018 | 2.29E-01 | 2.84E-01 | | 2.67E-01 | 4.54E-01 |
| 2019 | 2.05E-01 | 2.46E-01 | | 2.31E-01 | 4.32E-01 |
| 2020 | 2.25E-01 | 2.32E-01 | | 2.20E-01 | 4.83E-01 |

| Table S2 Gini coefficient of ECR by geographical area | | | | |
| --- | --- | --- | --- | --- |
| Year | Elderly care institutions | Active employees | Beds | Government financial allocation |
| 2009 | 6.79E-01 | 7.34E-01 | 7.44E-01 | 7.50E-01 |
| 2010 | 6.77E-01 | 7.40E-01 | 7.37E-01 | 7.42E-01 |
| 2011 | 6.73E-01 | 7.40E-01 | 7.29E-01 | 7.41E-01 |
| 2012 | 6.79E-01 | 7.37E-01 | 7.28E-01 | 7.46E-01 |
| 2013 | 6.78E-01 | 7.34E-01 | 7.26E-01 | 7.35E-01 |
| 2014 | 6.84E-01 | 7.33E-01 | 7.21E-01 | 6.72E-01 |
| 2015 | 6.81E-01 | 7.32E-01 | 7.14E-01 | 6.83E-01 |
| 2016 | 6.82E-01 | 7.34E-01 | 7.19E-01 | 7.11E-01 |
| 2017 | 6.80E-01 | 7.28E-01 | 7.20E-01 | 5.99E-01 |
| 2018 | 6.84E-01 | 7.35E-01 | 7.21E-01 | 7.44E-01 |
| 2019 | 6.84E-01 | 7.32E-01 | 7.17E-01 | 7.66E-01 |
| 2020 | 6.88E-01 | 7.29E-01 | 7.19E-01 | 7.91E-01 |

| Table S3 Theil Index of ECR allocation in China from 2009-2020 | | | | | | | | | | | | | | |
| --- | --- | --- | --- | --- | --- | --- | --- | --- | --- | --- | --- | --- | --- | --- |
| Year | Theil index | | | |  | The contribution rate of intraregion | | | |  | The contribution rate of interregion | | | |
|  | Elderly care institutions | Active employees | Beds | Government financial allocation |  | Elderly care institutions | Active employees | Beds | Government financial allocation |  | Elderly care institutions | Active employees | Beds | Government financial allocation |
| 2009 | 0.05485 | 0.11927 | 0.12174 | 0.44248 |  | 76.52% | 74.04% | 75.83% | 84.28% |  | 23.48% | 25.96% | 24.17% | 15.72% |
| 2010 | 0.05194 | 0.10878 | 0.10499 | 0.32114 |  | 69.08% | 73.52% | 86.48% | 90.99% |  | 30.92% | 26.48% | 13.52% | 9.01% |
| 2011 | 0.05112 | 0.10411 | 0.08981 | 0.38582 |  | 70.94% | 70.97% | 88.76% | 69.87% |  | 29.06% | 29.03% | 11.24% | 30.13% |
| 2012 | 0.04413 | 0.09956 | 0.08878 | 0.13997 |  | 82.08% | 70.09% | 90.53% | 54.96% |  | 17.92% | 29.91% | 9.47% | 45.04% |
| 2013 | 0.04412 | 0.08995 | 0.08332 | 0.30799 |  | 78.54% | 69.35% | 90.70% | 70.32% |  | 21.46% | 30.65% | 9.30% | 29.68% |
| 2014 | 0.08295 | 0.11444 | 0.11520 | 0.23516 |  | 91.47% | 71.28% | 92.32% | 63.24% |  | 8.53% | 28.72% | 7.68% | 36.76% |
| 2015 | 0.07349 | 0.12922 | 0.11129 | 0.18423 |  | 92.23% | 68.05% | 88.31% | 60.88% |  | 7.77% | 31.95% | 11.69% | 39.12% |
| 2016 | 0.08746 | 0.13784 | 0.11862 | 0.32420 |  | 83.87% | 65.68% | 84.41% | 91.45% |  | 16.13% | 34.32% | 15.59% | 8.55% |
| 2017 | 0.08477 | 0.13795 | 0.12220 | 0.46987 |  | 79.82% | 65.23% | 83.58% | 78.85% |  | 20.18% | 34.77% | 16.42% | 21.15% |
| 2018 | 0.08779 | 0.14758 | 0.11281 | 0.38613 |  | 70.19% | 66.11% | 83.98% | 82.04% |  | 29.81% | 33.89% | 16.02% | 17.96% |
| 2019 | 0.07201 | 0.11140 | 0.08265 | 0.35689 |  | 56.94% | 67.64% | 80.46% | 84.21% |  | 43.06% | 32.36% | 19.54% | 15.79% |
| 2020 | 0.08043 | 0.09503 | 0.07475 | 0.42691 |  | 40.55% | 68.76% | 74.56% | 86.92% |  | 59.45% | 31.24% | 25.44% | 13.08% |

Table S4 Contribution to the overall differences between regions from 2009 to 2020

| Year | Elderly care institutions | | | |  | Active employees | | | |  | Beds | | | |  | Government financial allocation | | | |
| --- | --- | --- | --- | --- | --- | --- | --- | --- | --- | --- | --- | --- | --- | --- | --- | --- | --- | --- | --- |
|  | NR | ER | CR | WR |  | NR | ER | CR | WR |  | NR | ER | CR | WR |  | NR | ER | CR | WR |
| 2009 | 3.16% | 4.60% | 6.79% | 58.72% |  | 3.32% | 44.28% | 7.29% | 15.02% |  | 1.33% | 33.30% | 14.09% | 32.22% |  | 5.98% | 33.30% | 23.08% | 19.89% |
| 2010 | 4.58% | 4.29% | 6.37% | 49.77% |  | 4.15% | 45.76% | 4.30% | 14.64% |  | 0.97% | 41.53% | 9.52% | 40.95% |  | 5.48% | 37.02% | 30.60% | 18.63% |
| 2011 | 5.11% | 6.53% | 6.88% | 48.45% |  | 3.63% | 44.21% | 4.03% | 13.52% |  | 0.12% | 41.51% | 10.33% | 42.37% |  | 4.23% | 30.59% | 13.35% | 15.63% |
| 2012 | 8.13% | 11.21% | 8.75% | 52.10% |  | 4.69% | 41.72% | 3.32% | 14.58% |  | 1.49% | 42.62% | 10.67% | 39.89% |  | 1.32% | 21.67% | 2.43% | 25.21% |
| 2013 | 8.56% | 31.11% | 7.30% | 34.05% |  | 5.63% | 40.67% | 2.65% | 14.81% |  | 1.78% | 41.85% | 10.70% | 39.40% |  | 2.46% | 23.00% | 9.17% | 29.23% |
| 2014 | 1.46% | 27.11% | 16.09% | 49.59% |  | 1.88% | 38.69% | 5.97% | 21.04% |  | 0.70% | 35.84% | 13.40% | 46.10% |  | 0.38% | 23.45% | 10.63% | 22.16% |
| 2015 | 2.65% | 30.57% | 24.23% | 36.80% |  | 1.85% | 39.86% | 6.19% | 12.11% |  | 1.24% | 37.72% | 19.68% | 30.72% |  | 1.66% | 23.99% | 10.67% | 19.45% |
| 2016 | 3.90% | 27.43% | 18.88% | 36.27% |  | 1.67% | 38.09% | 5.62% | 14.11% |  | 0.52% | 35.03% | 20.76% | 31.29% |  | 2.88% | 57.47% | 13.97% | 4.64% |
| 2017 | 1.94% | 27.39% | 16.89% | 37.62% |  | 2.17% | 38.73% | 5.68% | 11.18% |  | 0.23% | 36.58% | 20.52% | 28.84% |  | 0.16% | 44.77% | 3.04% | 11.14% |
| 2018 | 1.10% | 28.61% | 11.75% | 35.02% |  | 1.61% | 40.17% | 4.66% | 12.55% |  | 0.48% | 38.62% | 19.53% | 27.47% |  | 0.21% | 50.52% | 1.34% | 9.78% |
| 2019 | 0.89% | 25.67% | 6.81% | 30.70% |  | 2.56% | 41.57% | 1.99% | 15.33% |  | 0.51% | 42.61% | 13.00% | 26.23% |  | 0.05% | 54.31% | 0.50% | 10.24% |
| 2020 | 0.50% | 18.89% | 5.21% | 22.52% |  | 1.51% | 48.07% | 2.56% | 12.62% |  | 0.79% | 41.10% | 15.33% | 18.78% |  | 0.02% | 57.64% | 1.88% | 6.73% |

Table S5 Theil Index of ECR allocation among different regions from 2009 to 2020

| Year | Elderly care institutions | | | |  | Active employees | | | |  | Beds | | | |  | Government financial allocation | | | |
| --- | --- | --- | --- | --- | --- | --- | --- | --- | --- | --- | --- | --- | --- | --- | --- | --- | --- | --- | --- |
|  | NR | ER | CR | WR |  | NR | ER | CR | WR |  | NR | ER | CR | WR |  | NR | ER | CR | WR |
| 2009 | 0.0209 | 0.0069 | 0.0138 | 0.1154 |  | 0.0479 | 0.1435 | 0.0321 | 0.0642 |  | 0.0195 | 0.1102 | 0.0634 | 0.1406 |  | 0.3200 | 0.4005 | 0.3776 | 0.3155 |
| 2010 | 0.0290 | 0.0059 | 0.0124 | 0.0956 |  | 0.0549 | 0.1311 | 0.0175 | 0.0589 |  | 0.0124 | 0.1148 | 0.0373 | 0.1590 |  | 0.2142 | 0.3130 | 0.3672 | 0.2213 |
| 2011 | 0.0319 | 0.0088 | 0.0132 | 0.0916 |  | 0.0461 | 0.1208 | 0.0157 | 0.0521 |  | 0.0013 | 0.0979 | 0.0347 | 0.1408 |  | 0.1993 | 0.3098 | 0.1930 | 0.2232 |
| 2012 | 0.0441 | 0.0130 | 0.0145 | 0.0851 |  | 0.0574 | 0.1088 | 0.0124 | 0.0537 |  | 0.0162 | 0.0991 | 0.0355 | 0.1310 |  | 0.0227 | 0.0795 | 0.0128 | 0.1305 |
| 2013 | 0.0466 | 0.0359 | 0.0121 | 0.0556 |  | 0.0625 | 0.0957 | 0.0090 | 0.0493 |  | 0.0183 | 0.0912 | 0.0335 | 0.1214 |  | 0.0937 | 0.1852 | 0.1061 | 0.3330 |
| 2014 | 0.0150 | 0.0587 | 0.0502 | 0.1521 |  | 0.0268 | 0.1156 | 0.0257 | 0.0891 |  | 0.0100 | 0.1078 | 0.0580 | 0.1964 |  | 0.0111 | 0.1440 | 0.0939 | 0.1927 |
| 2015 | 0.0244 | 0.0586 | 0.0669 | 0.0999 |  | 0.0299 | 0.1345 | 0.0300 | 0.0578 |  | 0.0173 | 0.1096 | 0.0823 | 0.1262 |  | 0.0383 | 0.1154 | 0.0738 | 0.1323 |
| 2016 | 0.0432 | 0.0625 | 0.0621 | 0.1170 |  | 0.0291 | 0.1368 | 0.0291 | 0.0717 |  | 0.0078 | 0.1083 | 0.0925 | 0.1369 |  | 0.1182 | 0.4855 | 0.1703 | 0.0554 |
| 2017 | 0.0210 | 0.0604 | 0.0539 | 0.1175 |  | 0.0382 | 0.1390 | 0.0295 | 0.0568 |  | 0.0036 | 0.1163 | 0.0944 | 0.1298 |  | 0.0095 | 0.5473 | 0.0537 | 0.1928 |
| 2018 | 0.0124 | 0.0652 | 0.0388 | 0.1131 |  | 0.0305 | 0.1540 | 0.0259 | 0.0681 |  | 0.0069 | 0.1132 | 0.0829 | 0.1140 |  | 0.0105 | 0.5068 | 0.0195 | 0.1389 |
| 2019 | 0.0084 | 0.0479 | 0.0185 | 0.0813 |  | 0.0371 | 0.1200 | 0.0084 | 0.0628 |  | 0.0055 | 0.0913 | 0.0405 | 0.0797 |  | 0.0025 | 0.5024 | 0.0068 | 0.1343 |
| 2020 | 0.0058 | 0.0380 | 0.0162 | 0.0667 |  | 0.0206 | 0.1141 | 0.0094 | 0.0441 |  | 0.0085 | 0.0768 | 0.0443 | 0.0517 |  | 0.0012 | 0.6149 | 0.0310 | 0.1058 |


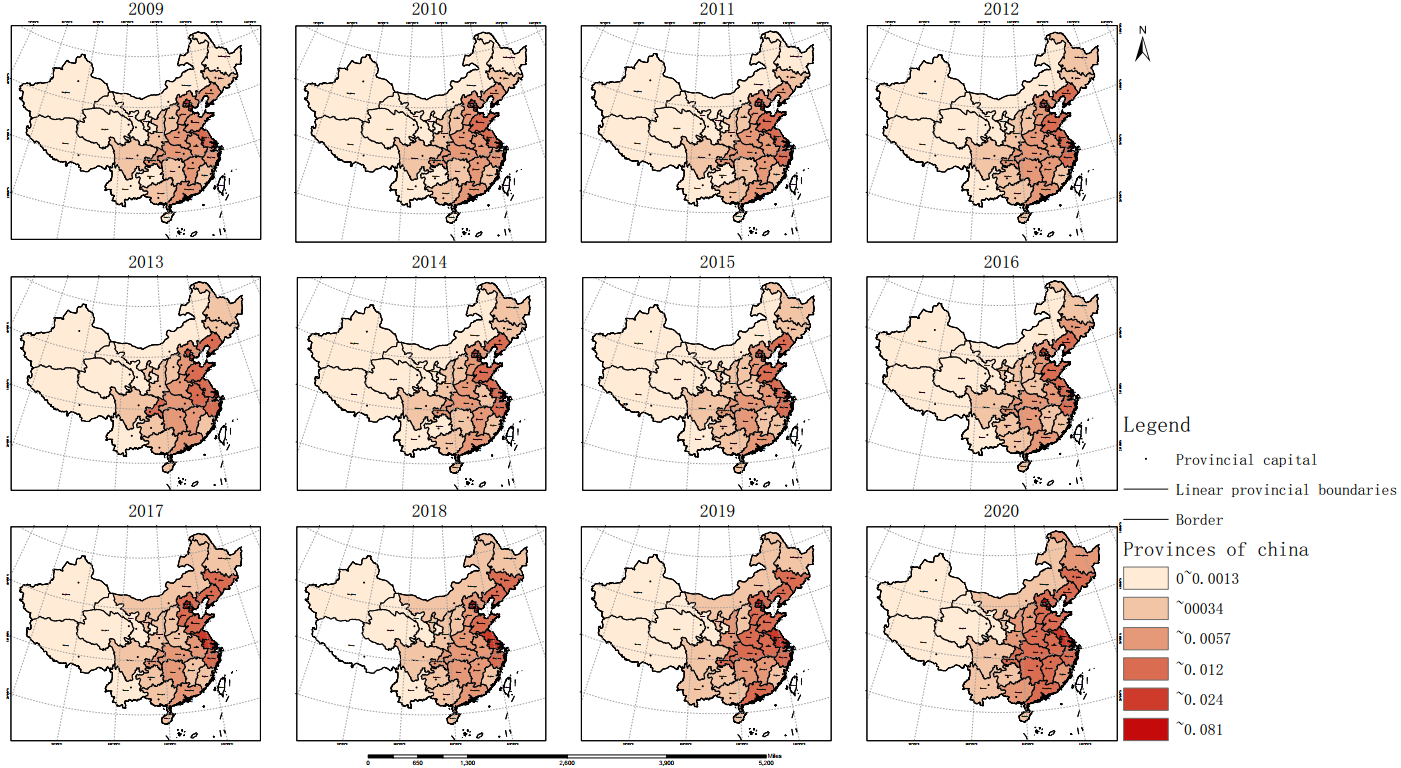


Figure S3 ORDI of Active employees


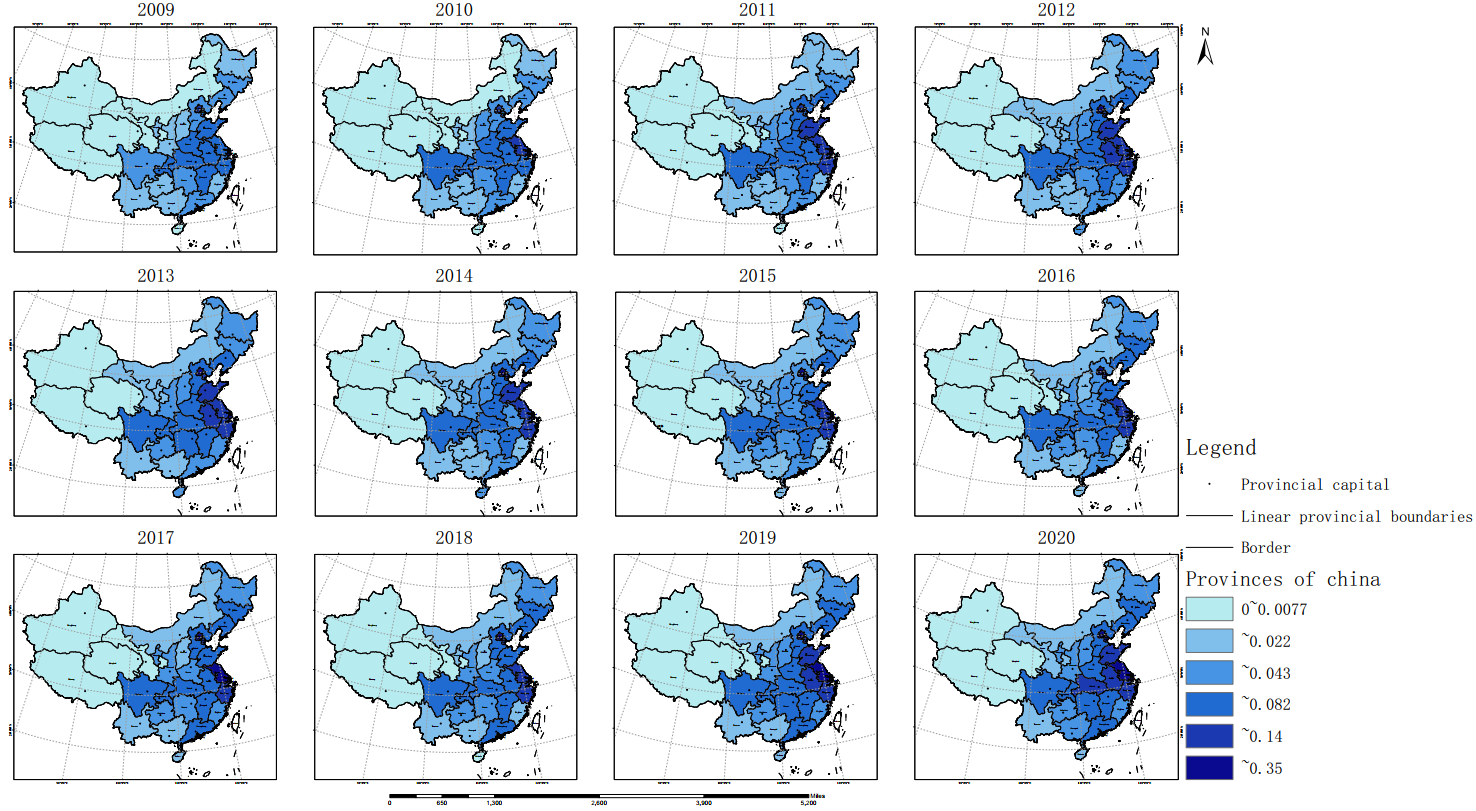


Figure S4 ORDI of Beds


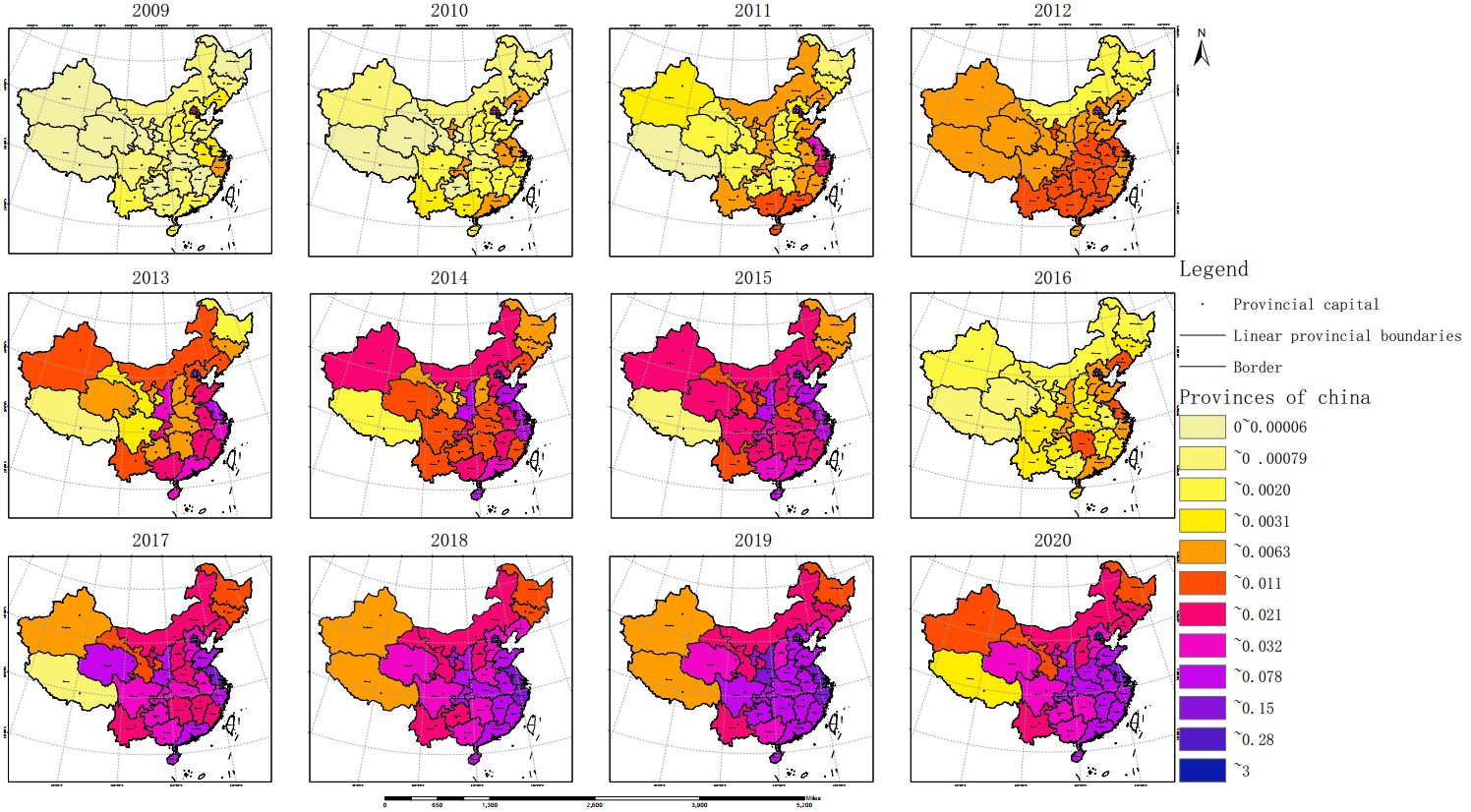


Figure S5 ORDI of Government financial allocation

Table S6 Descriptive statistics for inputs and outputs in China

| Year | items | input |  |  |  |  |  | output |  |  |  |  |  |
| --- | --- | --- | --- | --- | --- | --- | --- | --- | --- | --- | --- | --- | --- |
|  |  | I1 | I2 | I3 | I4 | I5 |  | O1 | O2 | O3 | O4 | O5 | O6 |
| 2009 | Average | 1280 | 8321 | 93219 | 1711281 | 124634 |  | 73411 | 17899828 | 58435 | 6739 | 9750 | 179653 |
|  | Max | 3111 | 20633 | 273077 | 5977014 | 466566 |  | 232137 | 78464972 | 206253 | 30063 | 146747 | 999964 |
|  | Min | 89 | 309 | 3778 | 99290 | 9067 |  | 2327 | 520468 | 1651 | 32 | 2 | 1 |
|  | St Dev | 904 | 6155 | 82710 | 1443800 | 105245 |  | 72008 | 18646345 | 63513 | 6678 | 27163 | 230640 |
| 2010 | Average | 1287 | 8828 | 101565 | 1609899 | 138990 |  | 78262 | 18902409 | 61227 | 11287 | 5345 | 236783 |
|  | Max | 3226 | 24013 | 309253 | 7327023 | 469206 |  | 243777 | 81553881 | 220169 | 33411 | 22467 | 1809563 |
|  | Min | 78 | 346 | 3875 | 15180 | 11691 |  | 2097 | 451660 | 1543 | 141 | 5 | 4447 |
|  | St Dev | 910 | 6686 | 90066 | 1571148 | 119707 |  | 75310 | 19349104 | 64145 | 9878 | 5770 | 379776 |
| 2011 | Average | 1318 | 9465 | 110377 | 2325389 | 179955 |  | 82834 | 21525938 | 63221 | 13414 | 6199 | 215415 |
|  | Max | 3258 | 26056 | 325003 | 8205620 | 555820 |  | 254736 | 77371432 | 217435 | 38319 | 20983 | 1147673 |
|  | Min | 77 | 442 | 4009 | 100621 | 7137 |  | 2222 | 779371 | 1430 | 176 | 3 | 15 |
|  | St Dev | 914 | 7095 | 94004 | 1937406 | 156994 |  | 76417 | 20425460 | 64194 | 10964 | 5697 | 294107 |
| 2012 | Average | 1429 | 10671 | 127989 | 2574894 | 214112 |  | 93551 | 22871332 | 65453 | 14604 | 7221 | 218607 |
|  | Max | 3386 | 29491 | 365270 | 8802045 | 710778 |  | 284643 | 82543367 | 225829 | 44380 | 30246 | 1074797 |
|  | Min | 79 | 513 | 7199 | 123740 | 10158 |  | 4131 | 776659 | 2049 | 418 | 5 | 15 |
|  | St Dev | 975 | 7972 | 106794 | 2141428 | 171422 |  | 82573 | 21027876 | 66340 | 12144 | 7576 | 290074 |
| 2013 | Average | 1370 | 11481 | 138552 | 2730937 | 234384 |  | 96154 | 23516457 | 67221 | 15904 | 7865 | 241950 |
|  | Max | 3336 | 30914 | 399449 | 9512571 | 790529 |  | 296923 | 73496113 | 232059 | 48926 | 28246 | 1720412 |
|  | Min | 78 | 601 | 7935 | 206747 | 19865 |  | 5800 | 1036537 | 3389 | 392 | 258 | 105 |
|  | St Dev | 942 | 8385 | 114237 | 2374556 | 196540 |  | 83945 | 20328512 | 66738 | 13151 | 7512 | 360859 |
| 2014 | Average | 1066 | 10756 | 125879 | 2600410 | 232409 |  | 82516 | 19937955 | 55502 | 14800 | 7606 | 208109 |
|  | Max | 3409 | 28752 | 385416 | 9077338 | 760714 |  | 307229 | 75293239 | 240348 | 52511 | 29134 | 955228 |
|  | Min | 80 | 627 | 6896 | 202545 | 23384 |  | 5055 | 885861 | 2574 | 440 | 105 | 350 |
|  | St Dev | 826 | 8027 | 106940 | 2472611 | 194547 |  | 75008 | 19145684 | 58095 | 12843 | 7542 | 271167 |
| 2015 | Average | 895 | 10266 | 115528 | 2316397 | 235744 |  | 69267 | 16875718 | 44347 | 12948 | 7600 | 261317 |
|  | Max | 2547 | 30717 | 388561 | 8484214 | 851817 |  | 227203 | 55464693 | 180009 | 46398 | 29471 | 1436994 |
|  | Min | 35 | 381 | 5022 | 123903 | 8897 |  | 1809 | 352678 | 1181 | 217 | 16 | 3605 |
|  | St Dev | 666 | 7787 | 94403 | 1991809 | 194072 |  | 58451 | 15447667 | 42880 | 10901 | 7905 | 368707 |
| 2016 | Average | 922 | 10929 | 122186 | 2517995 | 257208 |  | 70906 | 17344796 | 44035 | 14124 | 8463 | 237582 |
|  | Max | 2573 | 33463 | 410441 | 9205448 | 1008513 |  | 225115 | 56736655 | 174632 | 48365 | 32577 | 1187467 |
|  | Min | 7 | 91 | 1124 | 21678 | 17210 |  | 866 | 219725 | 815 | 22 | 29 | 3535 |
|  | St Dev | 685 | 8283 | 100554 | 2189834 | 220187 |  | 59136 | 15624820 | 42566 | 11789 | 8344 | 290379 |
| 2017 | Average | 928 | 11901 | 123694 | 1123500 | 59006 |  | 68108 | 17105917 | 11919 | 5777 | 5403 | 58313 |
|  | Max | 2588 | 34616 | 415143 | 4548938 | 277010 |  | 206997 | 56146165 | 39619 | 22215 | 24023 | 278411 |
|  | Min | 15 | 290 | 2776 | 11700 | 443 |  | 1680 | 203795 | 254 | 48 | 7 | 80 |
|  | St Dev | 669 | 8729 | 100235 | 1048225 | 66165 |  | 54176 | 14469607 | 11000 | 5205 | 5762 | 79575 |
| 2018 | Average | 925 | 12124 | 122378 | 2160765 | 277004 |  | 63733 | 16926180 | 38086 | 14854 | 10793 | 176114 |
|  | Max | 2248 | 36330 | 398617 | 7026724 | 946005 |  | 188823 | 52383996 | 126948 | 46787 | 36590 | 796040 |
|  | Min | 5 | 82 | 773 | 66635 | 24015 |  | 545 | 145910 | 320 | 74 | 151 | 556 |
|  | St Dev | 655 | 9044 | 98627 | 1606940 | 229233 |  | 50153 | 13709672 | 33695 | 11963 | 9427 | 204549 |
| 2019 | Average | 1109 | 14580 | 141544 | 2468708 | 375202 |  | 70171 | 18642450 | 39056 | 17194 | 13921 | 189932 |
|  | Max | 2549 | 43687 | 427268 | 6816500 | 1767492 |  | 195545 | 52254719 | 126414 | 49923 | 40584 | 844740 |
|  | Min | 8 | 136 | 3118 | 128617 | 15556 |  | 2181 | 379868 | 835 | 234 | 210 | 492 |
|  | St Dev | 788 | 10731 | 108924 | 1651363 | 355633 |  | 53102 | 14442229 | 33917 | 13057 | 11855 | 215743 |
| 2020 | Average | 1231 | 16716 | 157496 | 2678020 | 414751 |  | 71730 | 18949242 | 36310 | 19581 | 15839 | 198667 |
|  | Max | 3244 | 46882 | 442975 | 6856152 | 1235807 |  | 197916 | 50343268 | 114817 | 54535 | 47460 | 875785 |
|  | Min | 23 | 433 | 4160 | 107154 | 38714 |  | 2404 | 487017 | 1331 | 731 | 244 | 492 |
|  | St Dev | 897 | 12050 | 120978 | 1771223 | 316540 |  | 54048 | 14652302 | 31035 | 14899 | 12978 | 227062 |

Table S7 Efficiency values of ECR allocation in China by provincial-level

| No. | DMU | Overall Score | Rank | Term Efficiency | | | | | | | | | | | |
| --- | --- | --- | --- | --- | --- | --- | --- | --- | --- | --- | --- | --- | --- | --- | --- |
|  |  |  |  | Term1 | Term2 | Term3 | Term4 | Term5 | Term6 | Term7 | Term8 | Term9 | Term10 | Term11 | Term12 |
| 1 | Beijing | 0.9731 | 13 | 1 | 0.6768 | 1 | 1 | 1 | 1 | 1 | 1 | 1 | 1 | 1 | 1 |
| 2 | Tianjin | 1 | 1 | 1 | 1 | 1 | 1 | 1 | 1 | 1 | 1 | 1 | 1 | 1 | 1 |
| 3 | Hebei | 0.7889 | 27 | 1 | 1 | 1 | 0.8147 | 0.8565 | 0.7341 | 0.6242 | 0.5996 | 0.5812 | 0.7255 | 0.6938 | 0.837 |
| 4 | Shanxi | 0.6133 | 31 | 0.7912 | 0.6519 | 0.5635 | 0.5413 | 0.6223 | 0.5912 | 0.5624 | 0.5299 | 0.5366 | 0.635 | 0.6568 | 0.678 |
| 5 | Neimenggu | 0.8641 | 22 | 1 | 1 | 0.9471 | 0.8419 | 0.7598 | 0.8535 | 0.7139 | 0.6338 | 1 | 0.7779 | 0.8408 | 1 |
| 6 | Liaoning | 0.8591 | 23 | 1 | 1 | 0.6427 | 0.705 | 0.7996 | 0.6934 | 0.8469 | 0.7812 | 1 | 0.8405 | 1 | 1 |
| 7 | Jilin | 0.9627 | 15 | 1 | 0.9196 | 1 | 1 | 0.9042 | 1 | 0.7287 | 1 | 1 | 1 | 1 | 1 |
| 8 | Heilongjiang | 1 | 1 | 1 | 1 | 1 | 1 | 1 | 1 | 1 | 1 | 1 | 1 | 1 | 1 |
| 9 | Shanghai | 1 | 1 | 1 | 1 | 1 | 1 | 1 | 1 | 1 | 1 | 1 | 1 | 1 | 1 |
| 10 | Jiangsu | 1 | 1 | 1 | 1 | 1 | 1 | 1 | 1 | 1 | 1 | 1 | 1 | 1 | 1 |
| 11 | Zhejiang | 0.9611 | 16 | 1 | 0.7992 | 1 | 1 | 0.9536 | 1 | 0.8448 | 1 | 1 | 0.9359 | 1 | 1 |
| 12 | Anhui | 0.9356 | 18 | 0.8744 | 0.894 | 1 | 1 | 1 | 0.8203 | 0.8607 | 0.7773 | 1 | 1 | 1 | 1 |
| 13 | Fujian | 0.7317 | 29 | -0.9428 | 0.6064 | 0.6156 | 0.4959 | 0.6595 | 0.7519 | 0.6686 | 0.6233 | 1 | 0.8401 | 0.7914 | 1 |
| 14 | Jiangxi | 0.9886 | 9 | 1 | 1 | 1 | 1 | 1 | 1 | 0.863 | 1 | 1 | 1 | 1 | 1 |
| 15 | Shandong | 0.9857 | 10 | 1 | 1 | 1 | 1 | 1 | 1 | 1 | 1 | 1 | 0.8288 | 1 | 1 |
| 16 | Henan | 0.9316 | 19 | 1 | 1 | 1 | 1 | 1 | 1 | 0.8272 | 0.7768 | 0.827 | 0.8877 | 0.8609 | 1 |
| 17 | Hubei | 0.9935 | 8 | 1 | 1 | 1 | 1 | 1 | 1 | 1 | 1 | 1 | 1 | 1 | 0.9224 |
| 18 | Hunan | 0.9128 | 21 | 0.7546 | 1 | 0.8168 | 0.8314 | 1 | 1 | 1 | 0.7867 | 0.7642 | 1 | 1 | 1 |
| 19 | Guangdong | 1 | 1 | 1 | 1 | 1 | 1 | 1 | 1 | 1 | 1 | 1 | 1 | 1 | 1 |
| 20 | Guangxi | 0.822 | 24 | 0.7096 | 0.7285 | 1 | 0.7199 | 0.8355 | 1 | 0.7931 | 0.8318 | 1 | 1 | 0.6239 | 0.6214 |
| 21 | Hainan | 0.9734 | 12 | 1 | 1 | 1 | 0.6812 | 1 | 1 | 1 | 1 | 1 | 1 | 0.9998 | 1 |
| 22 | Chongqing | 0.9238 | 20 | 0.7681 | 0.6458 | 1 | 0.8082 | 0.8638 | 1 | 1 | 1 | 1 | 1 | 1 | 1 |
| 23 | Sichuan | 1 | 1 | 1 | 1 | 1 | 1 | 1 | 1 | 1 | 1 | 1 | 1 | 1 | 1 |
| 24 | Guizhou | 0.7731 | 28 | 0.523 | 0.6253 | 1 | 1 | 1 | 0.5934 | 1 | 0.6656 | 0.8089 | 0.6862 | 0.6633 | 0.7119 |
| 25 | Yunnan | 0.7907 | 26 | 0.6266 | 1 | 1 | 0.7053 | 1 | 0.7488 | 1 | 0.8787 | 0.6001 | 0.6359 | 0.6509 | 0.6418 |
| 26 | Xizang | 1 | 1 | 1 | 1 | 1 | 1 | 1 | 1 | 1 | 1 | 1 | 1 | 1 | 1 |
| 27 | Shaanxi | 0.9638 | 14 | 0.7659 | 1 | 1 | 1 | 1 | 1 | 1 | 1 | 1 | 0.8 | 1 | 1 |
| 28 | Gansu | 0.6992 | 30 | 1 | 0.6711 | 0.654 | 0.6137 | 0.6261 | 1 | 0.6353 | 0.5691 | 0.5022 | 0.6515 | 0.7536 | 0.7141 |
| 29 | Qinghai | 0.9481 | 17 | 1 | 1 | 1 | 1 | 1 | 1 | 1 | 0.708 | 0.669 | 1 | 1 | 1 |
| 30 | Ningxia | 0.9824 | 11 | 1 | 1 | 1 | 1 | 1 | 1 | 1 | 1 | 0.7886 | 1 | 1 | 1 |
| 31 | Xinjiang | 0.8158 | 25 | 0.811 | 0.893 | 0.6244 | 0.679 | 0.7558 | 0.7587 | 0.8325 | 0.6958 | 1 | 0.7388 | 1 | 1 |

| Table S8 Statistical description of Balance evaluation between efficiency and equity | | | | | | | | | | | | | | |
| --- | --- | --- | --- | --- | --- | --- | --- | --- | --- | --- | --- | --- | --- | --- |
|  |  | year |  |  |  |  |  |  |  |  |  |  |  | Total |
|  |  | 2010 | 2011 | 2012 | 2013 | 2014 | 2015 | 2016 | 2017 | 2018 | 2019 | 2020 | 2021 |  |
| 1 | Counts | 8 | 7 | 6 | 9 | 7 | 6 | 9 | 11 | 9 | 10 | 9 | 6 | 97 |
|  | % | 25.8% | 22.6% | 19.4% | 29.0% | 22.6% | 19.4% | 29.0% | 35.5% | 29.0% | 32.3% | 29.0% | 19.4% | 26.1% |
| 2 | Counts | 11 | 11 | 12 | 8 | 10 | 9 | 10 | 6 | 10 | 7 | 8 | 11 | 113 |
|  | % | 35.5% | 35.5% | 38.7% | 25.8% | 32.3% | 29.0% | 32.3% | 19.4% | 32.3% | 22.6% | 25.8% | 35.5% | 30.4% |
| 3 | Counts | 2 | 4 | 1 | 3 | 4 | 3 | 4 | 3 | 0 | 3 | 1 | 1 | 29 |
|  | % | 6.5% | 12.9% | 3.2% | 9.7% | 12.9% | 9.7% | 12.9% | 9.7% | 0.0% | 9.7% | 3.2% | 3.2% | 7.8% |
| 4 | Counts | 10 | 9 | 12 | 11 | 10 | 13 | 8 | 11 | 12 | 11 | 13 | 13 | 133 |
|  | % | 32.3% | 29.0% | 38.7% | 35.5% | 32.3% | 41.9% | 25.8% | 35.5% | 38.7% | 35.5% | 41.9% | 41.9% | 35.8% |
|  | Counts | 31 | 31 | 31 | 31 | 31 | 31 | 31 | 31 | 31 | 31 | 31 | 31 | 372 |
|  | % | 100.0% | 100.0% | 100.0% | 100.0% | 100.0% | 100.0% | 100.0% | 100.0% | 100.0% | 100.0% | 100.0% | 100.0% | 100.0% |
| Note:1- low-equity and low-efficiency ；2- low-equity and high-efficiency ；3- high-equity and low-efficiency ；  4- high-equity and high-efficiency | | | | | | | | | | | | | | |
